# Aberrant miR-378 expression promotes hepatic lipid accumulation via hijacking the bile acid-regulated autophagy

**DOI:** 10.1093/lifemeta/loaf038

**Published:** 2025-11-10

**Authors:** Zhoumin Niu, Ying Yan, Wei Liu, Qiuming Yao, Jingjing Chen, Siyi Shen, Jing Yu, Mei Ma, Zhuoyang Li, Yuting Wu, Yan Li, Cheng Hu, Hailuan Zeng, Xin Gao, Yuying Li, Jingjing Jiang, Hao Ying

**Affiliations:** Shanghai Institute of Nutrition and Health, University of Chinese Academy of Sciences, Chinese Academy of Sciences, Shanghai 200031, and Shanghai Jiao Tong University Affiliated Sixth People's Hospital, Shanghai 200233, China; Innovation Center for Intervention of Chronic Disease and Promotion of Health, Shanghai Institute of Nutrition and Health, Chinese Academy of Sciences, Shanghai 200031, China; Shanghai Institute of Nutrition and Health, University of Chinese Academy of Sciences, Chinese Academy of Sciences, Shanghai 200031, and Shanghai Jiao Tong University Affiliated Sixth People's Hospital, Shanghai 200233, China; Shanghai Institute of Nutrition and Health, University of Chinese Academy of Sciences, Chinese Academy of Sciences, Shanghai 200031, and Shanghai Jiao Tong University Affiliated Sixth People's Hospital, Shanghai 200233, China; Department of Endocrinology and Metabolism, Zhongshan Hospital, Fudan University, Shanghai 200031, China; Fudan Institute for Metabolic Diseases, Fudan University, Shanghai 200031, China; Shanghai Institute of Nutrition and Health, University of Chinese Academy of Sciences, Chinese Academy of Sciences, Shanghai 200031, and Shanghai Jiao Tong University Affiliated Sixth People's Hospital, Shanghai 200233, China; Shanghai Institute of Nutrition and Health, University of Chinese Academy of Sciences, Chinese Academy of Sciences, Shanghai 200031, and Shanghai Jiao Tong University Affiliated Sixth People's Hospital, Shanghai 200233, China; Shanghai Institute of Nutrition and Health, University of Chinese Academy of Sciences, Chinese Academy of Sciences, Shanghai 200031, and Shanghai Jiao Tong University Affiliated Sixth People's Hospital, Shanghai 200233, China; Shanghai Institute of Nutrition and Health, University of Chinese Academy of Sciences, Chinese Academy of Sciences, Shanghai 200031, and Shanghai Jiao Tong University Affiliated Sixth People's Hospital, Shanghai 200233, China; Shanghai Institute of Nutrition and Health, University of Chinese Academy of Sciences, Chinese Academy of Sciences, Shanghai 200031, and Shanghai Jiao Tong University Affiliated Sixth People's Hospital, Shanghai 200233, China; Shanghai Institute of Nutrition and Health, University of Chinese Academy of Sciences, Chinese Academy of Sciences, Shanghai 200031, and Shanghai Jiao Tong University Affiliated Sixth People's Hospital, Shanghai 200233, China; State Key Laboratory of Food Science and Resource, School of Food Science and Technology, Jiangnan University, Wuxi, Jiangsu 214122, China; Shanghai Diabetes Institute, Shanghai Key Laboratory of Diabetes Mellitus, Shanghai Clinical Centre for Diabetes, Shanghai Sixth People’s Hospital Affiliated to Shanghai Jiao Tong University School of Medicine, Shanghai 200233, China; Department of Endocrinology and Metabolism, Zhongshan Hospital, Fudan University, Shanghai 200031, China; Fudan Institute for Metabolic Diseases, Fudan University, Shanghai 200031, China; Department of Endocrinology and Metabolism, Zhongshan Hospital, Fudan University, Shanghai 200031, China; Fudan Institute for Metabolic Diseases, Fudan University, Shanghai 200031, China; Shanghai Institute of Nutrition and Health, University of Chinese Academy of Sciences, Chinese Academy of Sciences, Shanghai 200031, and Shanghai Jiao Tong University Affiliated Sixth People's Hospital, Shanghai 200233, China; Innovation Center for Intervention of Chronic Disease and Promotion of Health, Shanghai Institute of Nutrition and Health, Chinese Academy of Sciences, Shanghai 200031, China; Department of Endocrinology and Metabolism, Zhongshan Hospital, Fudan University, Shanghai 200031, China; Fudan Institute for Metabolic Diseases, Fudan University, Shanghai 200031, China; Shanghai Institute of Nutrition and Health, University of Chinese Academy of Sciences, Chinese Academy of Sciences, Shanghai 200031, and Shanghai Jiao Tong University Affiliated Sixth People's Hospital, Shanghai 200233, China; Innovation Center for Intervention of Chronic Disease and Promotion of Health, Shanghai Institute of Nutrition and Health, Chinese Academy of Sciences, Shanghai 200031, China; Key Laboratory of Food Safety Risk Assessment, Ministry of Health, Beijing 100021, China

**Keywords:** bile acid, FXR, miR-378, autophagy, lipid accumulation

## Abstract

Dysregulated autophagy contributes to liver steatosis, yet its regulation under distinct metabolic contexts remains poorly defined. Here, we identify bile acids (BAs) as critical modulators of hepatic autophagy. Circulating BA levels are elevated in human subjects with liver steatosis and independently associated with increased hepatic steatosis risk. High-fat diet (HFD) feeding increases circulating BA levels, while simultaneously reducing hepatic autophagic flux in mice, whereas pharmacological inhibition of farnesoid X receptor (FXR) enhances autophagy and alleviates steatosis in the livers of HFD-fed mice. Mechanistically, circulating BAs promote hepatic acetyl-CoA production through FXR-induced acyl-CoA oxidase 1 (ACOX1), which in turn suppresses autophagy by increasing the mechanistic target of rapamycin complex 1 (mTORC1) signaling. Similar to HFD feeding, prolonged fasting elevates BA levels and hepatic lipid accumulation, while concurrently upregulating hepatic miR-378, a positive regulator of BA synthesis. Although miR-378 exerts a cell-autonomous pro-autophagic effect during short-term fasting, it paradoxically drives lipid accumulation by suppressing hepatic autophagy via BA/FXR/ACOX1/acetyl-CoA axis in a non-cell-autonomous manner during either HFD feeding or prolonged fasting when BA action becomes considerable. Together, our study uncovers BAs as a previously unrecognized class of inhibitors of hepatic autophagy during prolonged fasting and in metabolic dysfunction-associated steatotic liver disease (MASLD), providing novel insights into context-dependent autophagic regulation of hepatic lipid metabolism and potential therapeutic strategies for MASLD.

## Introduction

Metabolic dysfunction-associated steatotic liver disease (MASLD), also known as non-alcoholic fatty liver disease (NAFLD) [1], is the most widespread chronic liver disease and continues to increase in prevalence [2]. MASLD is a spectrum of metabolic conditions ranging from benign lipid accumulation to severe conditions [3]. A crucial feature of MASLD lies in the disruption of lipid homeostasis, which leads to an excessive accumulation of fatty acids in the liver, ultimately manifesting as intracellular lipid droplets (LDs) [4]. Developing ­pharmacological strategies specifically aimed at managing hepatic steatosis may prevent or reverse the progression of MASLD [5–7].

Autophagy is an essential lysosomal degradation process involving breaking down various cellular components, which is often triggered in response to cellular stress, such as fasting, and plays a pivotal role in intracellular homeostasis [8]. Lipophagy, as one kind of autophagy involving the breakdown of LDs within the cells, is able to serve as a vital means of maintaining lipid homeostasis [9]. Increasing evidence suggests that dysregulation of lipophagy is closely associated with MASLD [10, 11]. Recently, it has been proposed that targeting hepatic autophagy may present a promising therapeutic approach for treating MASLD [12, 13].

Primary bile acids (BAs) are synthesized in the liver, stored in the gallbladder, and then released into the duodenum after a meal to facilitate the absorption of dietary fats and lipid-soluble vitamins. Afterwards, enterocytes reabsorb BAs and transport them back to the liver via the portal vein (PV) [14]. BAs can also serve as signaling molecules that regulate metabolic homeostasis through the activation of farnesoid X receptor (FXR), a nuclear BA receptor [15, 16]. Emerging evidence suggests that alterations in BA meta­bolism are associated with many metabolic diseases, including MASLD [17]. Recently, it has been suggested that BAs suppress hepatic autophagy at the transcriptional level via FXR [18, 19]. However, whether and how BA-regulated autophagy is involved in liver steatosis remains elusive.

Here, we found that elevated circulating total BA levels were not only observed in human subjects with hepatic steatosis but also independently associated with increased hepatic steatosis risk. Accordingly, high-fat diet (HFD)-fed mice displayed increased circulating BA levels with concurrent autophagic suppression, while inhibition of FXR by an antagonist could enhance autophagy and alleviate liver steatosis in HFD-fed mice. Mechanistic studies suggest that BAs are able to suppress hepatic autophagy through the mechanistic target of rapamycin complex 1 (mTORC1) signaling via BA/FXR/acyl-CoA oxidase 1 (ACOX1)/acetyl-CoA axis. Further studies indicate that either prolonged fasting or HFD feeding-induced elevation of hepatic microRNA-378 (miR-378), an upstream controller of circulating BA levels, can promote lipid accumulation via hijacking the intrinsic role of BAs in autophagy suppression. Thus, we uncovered novel physiological and pathological roles of BAs in the regulation of autophagy and lipid meta­bolism during prolonged fasting and in MASLD.

## Results

### Higher serum BA levels are independently associated with a higher risk of hepatic steatosis

**Figure 1:**
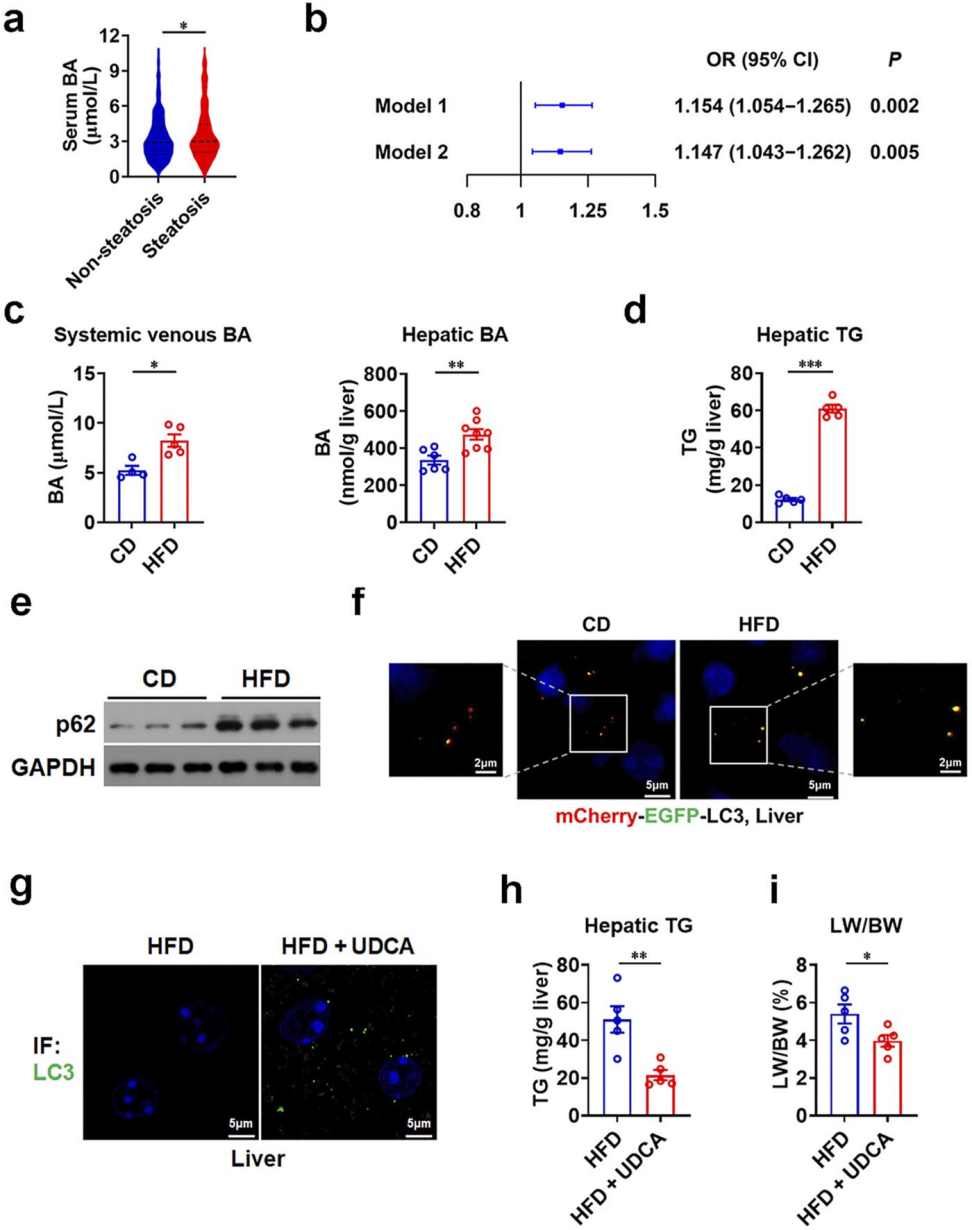
Elevated circulating BAs and defective autophagy are associated with hepatic steatosis in human subjects and HFD-fed mice. (a) Serum BA levels in subjects without hepatic steatosis (non-steatosis, *n *= 414) and with hepatic steatosis (steatosis, *n *= 194). (b) Logistic regression analysis of association between serum BA levels and hepatic steatosis risk in males. Model 1: adjusted for age; Model 2: further adjusted for TG and fasting blood glucose based on Model 1. OR: odds ratio; CI: confidence interval. (c) Systemic venous (*n *= 4−5) and hepatic (*n *= 6−8) BA levels in HFD-fed mice. (d) Liver TG levels of HFD-fed mice (*n *= 5). (e) Western blot analysis of p62 in the livers of HFD-fed mice. (f) Representative images of mCherry-EGFP-LC3 puncta in the livers of HFD-fed AFR mice. (g) LC3 immunofluorescence in the livers of HFD-fed mice treated with UDCA. (h and i) Liver TG levels (h) and liver-to-body weight ratio (i) in HFD-fed mice treated with UDCA (*n *= 5). Mean ± SEM are shown. *P* values were calculated by two-tailed unpaired Student’s *t*-test. ^*^P < 0.05; ^**^P < 0.01; ^***^P < 0.001.

In this study, we investigated a cohort of 608 subjects, including 414 subjects without hepatic steatosis and 194 subjects with hepatic steatosis, and observed that subjects with hepatic steatosis exhibited higher levels of serum BAs (Fig. 1a). Importantly, logistic regression analysis adjusted for age revealed that higher levels of circulating BAs were associated with a higher risk of hepatic stea­tosis (odds ratio [OR]: 1.154 per µmol/L increase; 95% confidence interval [CI]: 1.054–1.265; *P *= 0.002; Fig. 1b; model 1). More importantly, after further adjusting for triglycerides (TGs) and fasting blood glucose, higher levels of serum BAs were also associated with a higher risk of hepatic steatosis (OR: 1.147 per µmol/L increase; 95% CI: 1.043–1.262; *P *= 0.005; Fig. 1b; model 2). These results suggest that higher levels of serum BAs are independently associated with a higher risk of hepatic steatosis.

### HFD-induced liver steatosis in mice is accompanied by increased circulating BAs and impaired hepatic autophagy

In agreement with previous studies [20], systemic venous and hepatic BA levels were elevated in mice with hepatic steatosis induced by HFD feeding (Fig. 1c). Further analysis demonstrated that autophagy was impaired in the fatty livers of HFD-fed mice as evident from an accumulation of p62 protein (Fig. 1d and e; Supplementary Fig. S1a–c). Moreover, by taking advantage of ­autophagic flux reporter (AFR) mice with hepatic expression of mCherry-EGFP-LC3 reporter gene we developed recently ­(Supplementary  Fig. S1d), we observed a reduction of autophagic flux in the steatotic livers of HFD-fed mice, as evident from a decrease in the number of red LC3 puncta (Fig. 1f). Furthermore, we also observed a positive correlation between circulating BA levels and hepatic p62 levels, as quantified by immunostaining, in HFD-fed mice (Supplementary Fig. S1e), further supporting the notion that elevated BA levels are associated with impaired autophagy in the steatotic liver.

### Enhancement of autophagy by FXR inhibition improves liver steatosis in mice

It has been shown that activation of FXR can suppress hepatic autophagy [18, 19]. Given that the majority of BAs are FXR agonists and one of the physiological functions of BAs in the liver is to inhibit their own synthesis through FXR [14], based on the above data, we speculated that increased circulating BAs might lead to hepatic FXR activation, thereby suppressing autophagy in the liver. Thus, we then tested whether enhancement of autophagy by using ursodeoxycholic acid (UDCA), an FXR antagonist, could improve liver steatosis in HFD-fed mice. We found that UDCA treatment not only increased hepatic autophagic induction, as evident from the increased number of LC3 puncta (Fig. 1g) and elevated LC3 protein levels (Supplementary Fig. S1f) but also reduced the liver weight and hepatic TG content in HFD-fed mice (Fig. 1h and i), suggesting that enhancement of autophagy by FXR inhibition may contribute to the improvement of liver steatosis. Notably, under our experimental conditions, UDCA treatment did not alter the AMP-activated protein kinase (AMPK) signaling (Supplementary Fig. S1g). These results suggest that its pro-autophagic effects are primarily mediated via FXR antagonism, although other mechanisms cannot be excluded. Thus, we speculated that elevated BAs might serve as a causative factor for hepatic steatosis by activating FXR to promote lipid accumulation through the suppression of autophagy.

### Hepatic FXR activation inhibits autophagy through elevating acetyl-CoA levels

**Figure 2:**
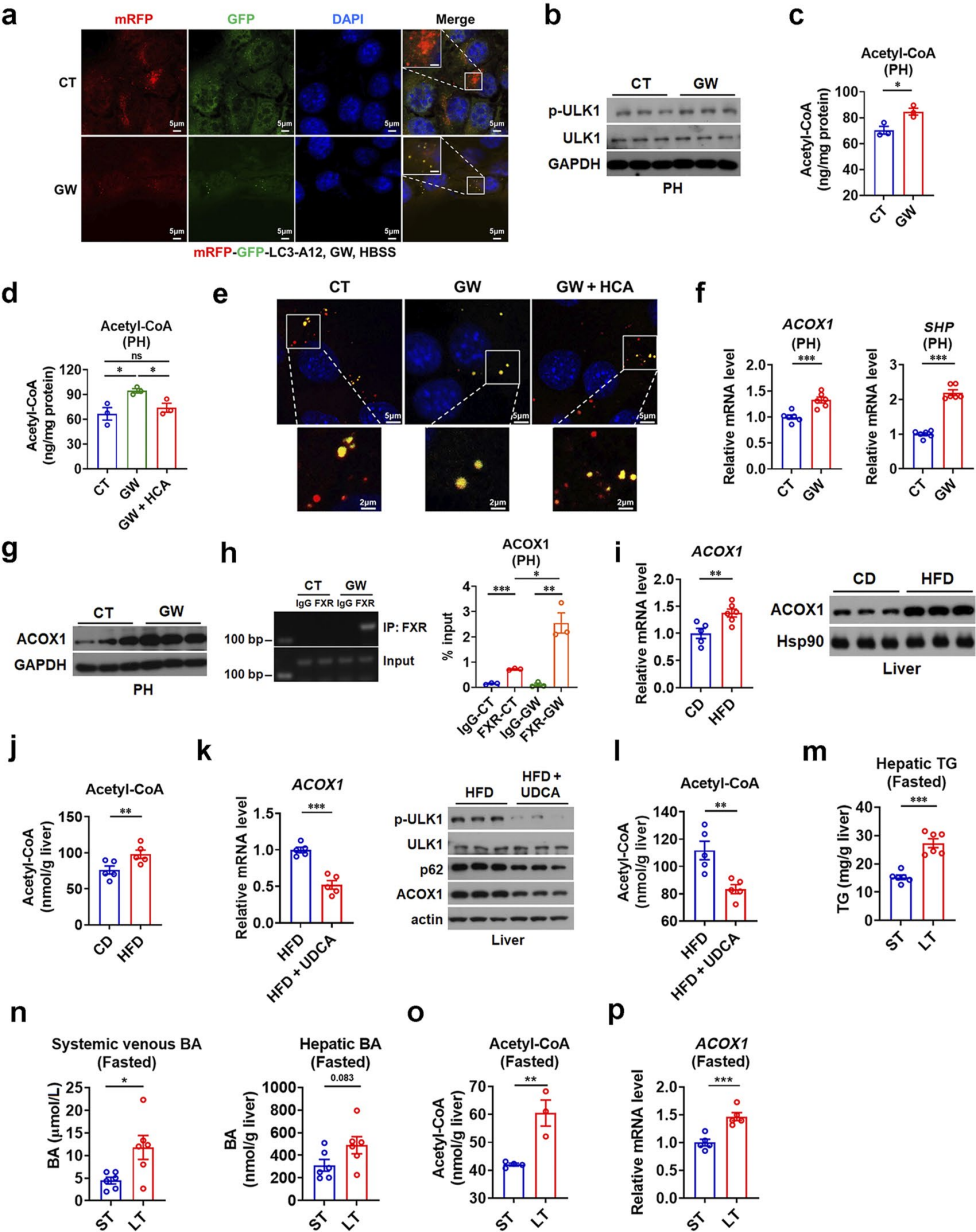
Hepatic FXR activation suppresses autophagy by increasing acetyl-CoA levels. (a) Representative images of AML12 cells stably expressing mRFP-GFP-LC3 treated with GW4064 and following HBSS starvation. AML12 cells stably expressing the autophagy reporter mRFP-GFP-LC3 were treated with GW4064, followed by 2 h of HBSS starvation, and stained with DAPI. Scale bars (original: 5 μm; enlarged: 2.5 μm) are shown in the figure. (b) Western blot analysis of p-ULK1 in PHs treated with GW4064. (c) Acetyl-CoA content in PHs treated with 2 µmol/L GW4064 (GW) for 24 h (*n *= 3). (d) Acetyl-CoA levels in PHs treated with vehicle, 2 μmol/L GW4064 (GW), or 2 μmol/L GW4064 in combination with 200 μmol/L HCA (*n *= 3). (e) Representative images of AML12 cells stably expressing mRFP-GFP-LC3 treated with GW4064 and following HBSS starvation. Ad-RFP-GFP-LC3-infected AML12 cells were treated with vehicle, 2 μmol/L GW4064 or 2 μmol/L GW4064 in combination with 200 μmol/L HCA, followed by 2 h of HBSS starvation. (f) Relative mRNA levels of *ACOX1* and *SHP* in PHs treated with GW (*n *= 6). (g) Western blot analysis of ACOX1 in PHs treated with GW. (h) ChIP-PCR analysis of FXR recruitment to the *ACOX1* promoter in PHs. (i) Relative mRNA levels (left, *n *= 5 − 6) and western blot analysis (right) of hepatic ACOX1 in HFD-fed mice. (j) Hepatic acetyl-CoA content in HFD-fed mice (*n *= 5). (k) Relative mRNA levels of hepatic *ACOX1* and western blot analysis of p-ULK1, p62, and ACOX1 in the livers of HFD-fed mice treated with UDCA (*n *= 5). (l) Hepatic acetyl-CoA content in HFD-fed mice treated with UDCA (*n *= 5). (m) Hepatic TG levels in mice after short-term or prolonged fasting as indicated (*n *= 6). (n) Systemic venous and hepatic total BA levels in mice after short-term or prolonged fasting as indicated (*n *= 6). (o) Acetyl-CoA content in mice after short-term or prolonged fasting as indicated (*n *= 3−4). (p) Relative mRNA levels of *ACOX1* in mice after short-term or prolonged fasting as indicated (*n *= 5). *P* values were calculated by two-tailed unpaired Student’s *t*-test. ^*^P < 0.05; ^**^P < 0.01; ^***^P < 0.001. ns, not significant.

Pharmacological activation of FXR has been reported to inhibit hepatic autophagy by repressing the transcription of autophagy-related genes [18, 19]. In agreement with the notion that FXR is a negative regulator of autophagy, inhibition of autophagic flux by the treatment of either the synthetic agonist GW4064 or the endogenous agonist chenodeoxycholic acid (CDCA) of FXR could be observed in hepatocytes upon autophagic induction (Fig. 2a; Supplementary Fig. S2a), while attenuated accumulation of LC3 puncta was observed after bafilomycin A1 (Baf-A1) treatment (Supplementary  Fig. S2b). Consistently, phosphorylation of Unc-51-like autophagy activating kinase 1 (ULK1) was increased following GW4064 treatment in primary hepatocytes (PHs) (Fig. 2b), indicating upregulation of mTORC1 signaling. However, GW4064 treatment could not alter the mRNA levels of autophagy-related genes in PHs, suggesting the involvement of alternative mechanisms (Supplementary Fig. S2c).

Interestingly, an approach using unbiased metabolomics (NODE: OEZ00021653) revealed that FXR activation in PHs by GW4064 ­treatment elevated the levels of acetyl-CoA, a vital metabolic cue that inhibits autophagy by activating mTORC1 signaling [21, 22] ­(Supplementary  Fig. S2d). This finding was subsequently validated by a targeted fluorescence-based assay (Fig. 2c). More interestingly, administration of the ATP-citrate lyase (ACLY) inhibitor, hydroxycitric acid (HCA), could reduce acetyl-CoA levels and restore autophagic flux (Fig. 2d and e), suggesting that the increased acetyl-CoA levels contribute to the anti-autophagic effect of FXR. Given that FXR is a central metabolic regulator, these results support the notion that acetyl-CoA serves as a downstream effector of FXR in regulating autophagy. Collectively, the above findings indicate that BAs may suppress autophagy via acetyl-CoA−mTOR signaling pathway.

To investigate the mechanism underlying FXR-induced acetyl-CoA elevation, we examined the expression of pyruvate dehydrogenase kinase isoenzyme 4 (PDK4), which inhibits the conversion of pyruvate to acetyl-CoA through the pyruvate dehydrogenase complex. However, we found that GW4064 treatment enhanced *PDK4* mRNA expression rather than reducing it, failing to justify the observed accumulation of acetyl-CoA (Supplementary Fig. S2e). We next assessed the expression of *ACLY* and acyl-CoA synthetase short-chain family member 2 (*ACSS2*), two additional enzymes in acetyl-CoA synthesis, and observed that GW4064 ­treatment had no significant impact on their mRNA levels ­(Supplementary  Fig. S2e). Consistent with the established role of ACOX1, a key enzyme in peroxisome-mediated acetyl-CoA production, in the regulation of autophagy [21], a subsequent transcriptional profiling employing RNA sequencing (RNA-seq) (NODE: OEP00006649) and validation by qPCR in GW4064-treated PHs collectively revealed that the repression of hepatic autophagy by FXR activation was accompanied by an increase in *ACOX1* expression (Fig. 2f). The regulation of ACOX1 by GW4064 treatment was confirmed at the protein level using western blotting (Fig. 2g). Similar results could be obtained when CDCA, an endogenous FXR agonist, was employed instead of GW4064 (Supplementary Fig. S2f and g). Importantly, our chromatin immunoprecipitation (ChIP) analysis revealed that FXR could be recruited to the promoter region of *ACOX1* (Fig. 2h), indicating that FXR regulates ACOX1 expression at the transcriptional level. Together, these data suggest that BAs and FXR can inhibit autophagy via a previously undescribed mechanism involving ACOX1-mediated acetyl-CoA production.

We also assessed the ACOX1 expression and hepatic acetyl-CoA content in HFD-fed mice with elevated BA levels. In line with the data from HFD-fed mice, increases in the mRNA levels of known FXR target genes, ACOX1 mRNA and protein levels, and acetyl-CoA levels were all observed in the livers of HFD-fed mice (Fig. 2i and j; Supplementary Fig. S2h). Accordingly, the mRNA levels of *ACLY* and *ACSS2* were not elevated in HFD-fed mice (Supplementary Fig. S2h). On the other hand, UDCA treatment reduced the mRNA levels of established FXR target genes, both the mRNA and protein levels of ACOX1, and the levels of p62 and p-ULK1 proteins, as well as the levels of acetyl-CoA in the livers of HFD-fed mice (Fig. 2k and l; Supplementary  Fig. S2i). These findings suggest that UDCA treatment might enhance mTORC1-controlled autophagy by targeting the FXR-ACOX1-mediated acetyl-CoA production, leading to a decrease in TG accumulation in these mice. Collectively, our above data support the conclusion that elevated circulating BAs promote hepatic steatosis by inhibiting autophagy.

As prolonged fasting (also referred to as long-term fasting) is known to induce physiological liver steatosis, we also tested whether circulating BA levels were also elevated and involved in the development of hepatic lipid accumulation after prolonged fasting. Interestingly, we observed that prolonged fasting induced hepatic lipid accumulation in mice concomitant with elevated systemic and hepatic BA levels (Fig. 2m and n). Moreover, hepatic acetyl-CoA content, *ACOX1* mRNA levels, and mRNA levels of documented FXR target genes were all increased following prolonged fasting (Fig. 2o and p; Supplementary Fig. S2j). Accordingly, the mRNA levels of *ACLY* and *ACSS2* were not elevated after prolonged fasting (Supplementary Fig. S2j). Collectively, we propose that BAs function as negative regulators of hepatic autophagy through FXR/ACOX1/acetyl-CoA axis to exacerbate hepatic steatosis during prolonged fasting.

### Hepatic miR-378 suppresses autophagy and promotes steatosis in the liver via a non-cell-autonomous BA-mediated mechanism upon prolonged fasting

**Figure 3:**
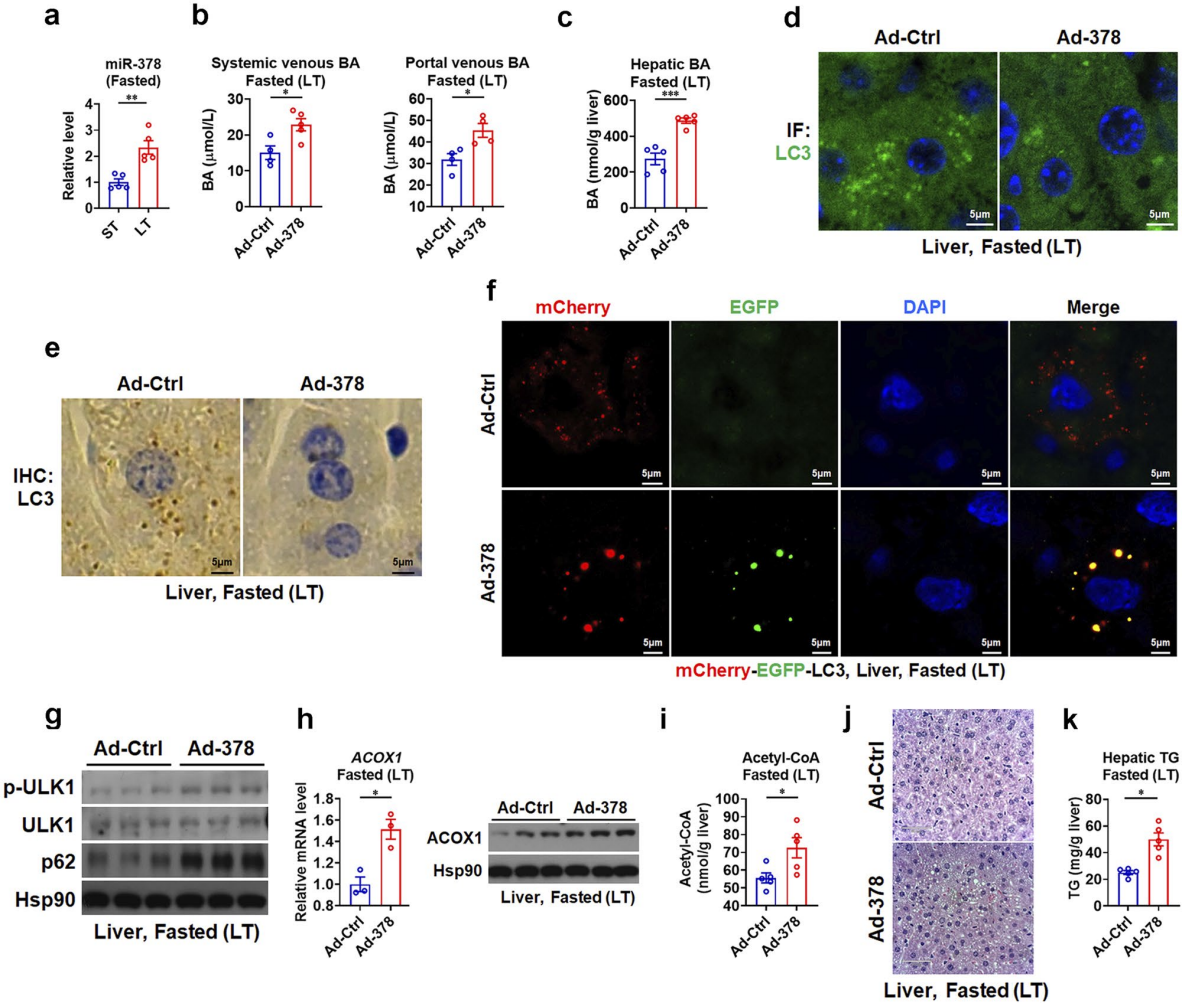
Hepatic miR-378 suppresses autophagy by increasing BA levels during prolonged fasting. (a) Relative miR-378 levels in the livers of mice after short-term or prolonged fasting as indicated (*n *= 5). (b) Systemic venous BAs (*n *= 4−5) and portal venous BAs (*n *= 4) in mice infected with Ad-378 after long-term fasting. (c) Hepatic total BAs in mice infected with Ad-378 after long-term fasting (*n *= 5). (d and e) Immunofluorescence (d) and immunohistochemistry (e) staining of LC3 in the livers of mice infected with Ad-378 after long-term fasting. (f) Representative images of mCherry-EGFP-LC3 puncta in the livers of AFR mice infected with Ad-378 after long-term fasting. (g) Western blot analysis of p-ULK1 and p62 in the livers of mice infected with Ad-378 after long-term fasting. (h) Relative mRNA levels (left) and western blot analysis (right) of hepatic ACOX1 in mice infected with Ad-378 after long-term fasting (*n *= 3). (i) Hepatic acetyl-CoA content in mice infected with Ad-378 after long-term fasting (*n *= 5). (j and k) H&E staining of liver sections (j) and liver TG levels (k) in mice infected with Ad-378 after long-term fasting (*n *= 5). Means ± SEM are shown. *P* values were calculated by two-tailed unpaired Student’s *t*-test. ^*^P < 0.05; ^**^P < 0.01; ^***^P < 0.001. ns, not significant.

As both HFD feeding and prolonged fasting could increase hepatic lipid content, while concurrently upregulating hepatic miR-378 (Fig. 3a; Supplementary Fig. S3a), a positive regulator of BA biosynthesis by targeting v-maf musculoaponeurotic fibrosarcoma oncogene homolog G (MAFG) [23–25], which we identified previously [26], we then tested whether miR-378 could affect hepatic lipid accumulation via BA-mediated autophagy suppression upon prolonged fasting. As expected, we found that overexpression of miR-378 in the livers of mice by adenoviral miR-378 (Ad-378) infection could elevate not only BA levels but also the expression of hepatic FXR target genes (Fig. 3b and c; Supplementary Fig. S3b and c). In agreement with our notion that BAs act as negative regulator of hepatic autophagy, both immunofluorescence and immunohistochemistry analyses revealed a decrease in the number of LC3 puncta in the livers of mice infected with Ad-378 (Fig. 3d and e). Consistently, decreased autophagic flux was observed in the livers of AFR mice infected with Ad-378 upon prolonged fasting, as evident from a decrease in the number of red LC3 puncta (Fig. 3f).

Accordingly, Ad-378 infection resulted in an increase in either the phosphorylation of ULK1 or the protein levels of p62 in the livers of mice under prolonged fasting conditions (Fig. 3g). Meanwhile, increased ACOX1 mRNA and protein levels and acetyl-CoA levels were observed in the livers of these Ad-378-infected mice after prolonged fasting (Fig. 3h and i). In line with these data, GW4064 treatment in PHs cultured in low-glucose medium supplemented with fatty acids, which mimics the nutritional status during prolonged fasting, led to increased expression of small ­heterodimer partner (*SHP*), an FXR target gene, as well as elevated ACOX1 mRNA and protein levels (Supplementary Fig. S3d and e). As exacerbated hepatic steatosis was found in the livers of mice infected with Ad-378 (Fig. 3j and k; Supplementary Fig. S3f–h), we speculated that hepatic miR-378 might act to promote lipid accumulation in the liver via a non-cell-autonomous mechanism involving BA/FXR/ACOX1/acetyl-CoA axis-mediated autophagy attenuation after prolonged fasting.

### Hepatic miR-378 can promote autophagy by targeting MAFG cell-autonomously

**Figure 4:**
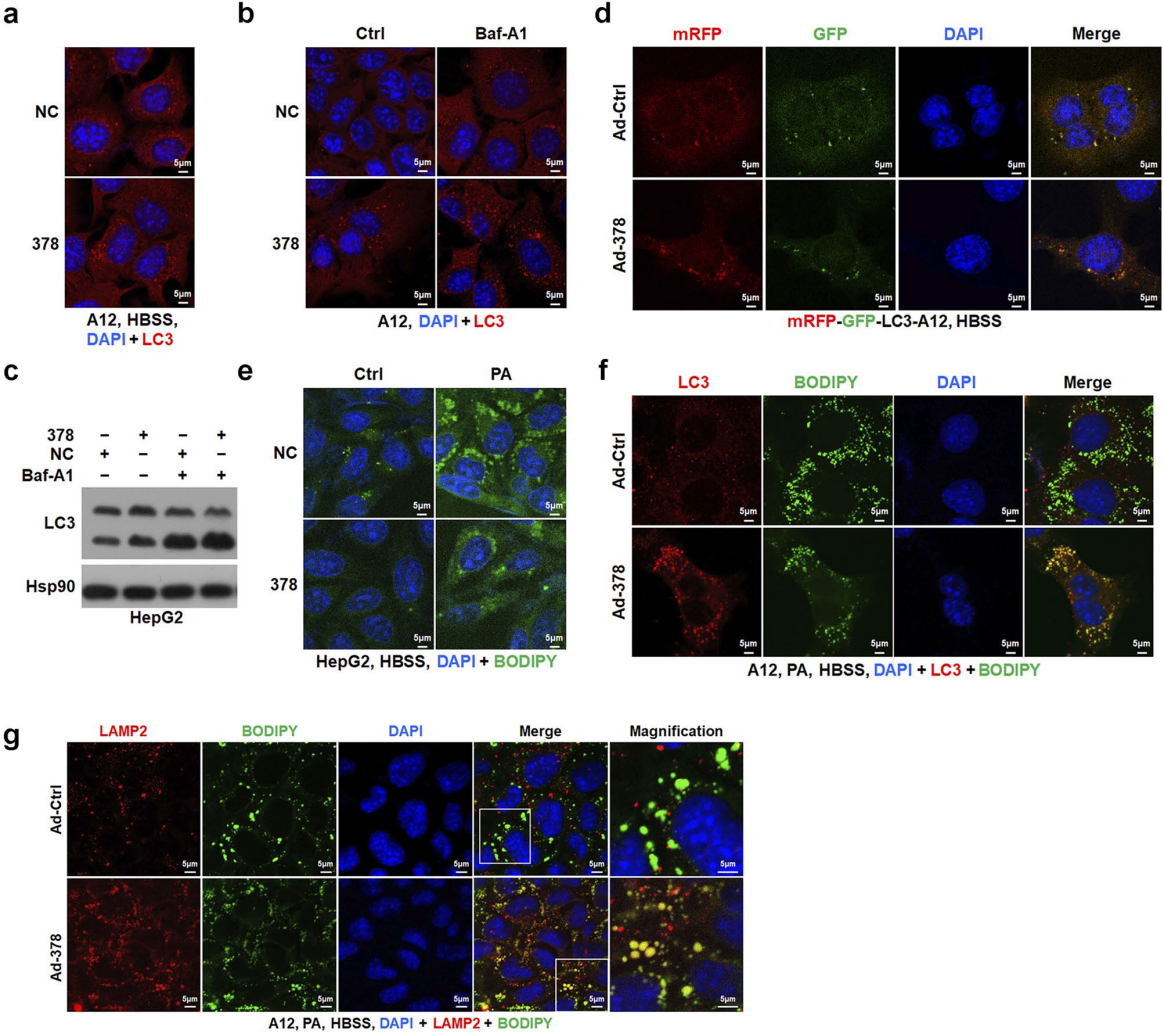
miR-378 overexpression promotes lipophagy in hepatocytes. (a) Immunofluorescence staining of LC3 in AML12 cells transfected with miR-378 mimics under HBSS starvation conditions. (b) Immunofluorescence staining of LC3 in AML12 cells transfected with miR-378 in the absence or presence of Baf-A1. (c) Western blot analysis of LC3 in HepG2 cells transfected with miR-378 mimics in the absence or presence of Baf-A1. (d) Representative images of AML12 cells stably expressing mRFP-GFP-LC3 infected with Ad-378 and following HBSS treatment. AML12 cells stably expressing the autophagy reporter mRFP-GFP-LC3 were infected with Ad-378, followed by HBSS treatment for 2 h. (e) BODIPY staining of HepG2 cells transfected with miR-378 mimics co-treated with or without PA followed by HBSS treatment. (f and g) Immunofluorescence staining for BODIPY (593/403) and LC3 (f) or LAMP2 (g) in PA-pretreated AML12 cells infected with Ad-378, followed by HBSS treatment.

Surprisingly, overexpression of miR-378 increased rather than inhibited autophagic flux in cultured hepatocytes, while CDCA administration attenuated the pro-autophagic effect of miR-378 upon autophagic induction (Supplementary Fig. S4a). Based on these *in vitro* data and the above *in vivo* data, we speculated that hepatic miR-378 might regulate autophagy in the mouse liver through both cell-autonomous and non-cell-autonomous mechanisms. As expected, transfection of miR-378 mimics resulted in an increase in the number of LC3 puncta upon autophagic induction by Hank’s balanced salt solution (HBSS) administration in AML12 hepatocytes (Fig. 4a). Moreover, increased accumulation of LC3 puncta was observed in AML12 cells transfected with miR-378 mimics after treatment of either Baf-A1 or chloroquine (CQ) (Fig. 4b; Supplementary Fig. S4b). Similar results were obtained by examining LC3 protein levels and lipidation in HepG2 cells transfected with miR-378 mimics in the presence of Baf-A1 (Fig. 4c). Consistently, attenuated LC3 lipidation was observed in HepG2 cells treated with miR-378 inhibitor in the presence of Baf-A1 ­(Supplementary  Fig. S4c). Downregulation of p-ULK1 was observed after treatment of miR-378 mimics in PHs (Supplementary Fig. S4d). The effect of miR-378 on autophagic flux was further verified by using AML12 hepatocytes stably expressing mRFP-GFP-LC3 reporter gene or infected with adenoviral RFP-GFP-LC3 (Ad-GFP-RFP-LC3) or PHs isolated from AFR mice (Fig. 4d; Supplementary Fig. S4e and f). Together, these *in vitro* data suggest that miR-378 is able to promote autophagic flux in hepatocytes in a cell-autonomous manner.

We further examined the interactions of LDs with autophagosomes and lysosomes by employing 4,4-difluoro-4-bora-3a, 4a-diaza-s-indacene (BODIPY) to image intracellular LDs. Transfection of miR-378 mimics reduced the number of LDs in HepG2 cells either with or without palmitic acid (PA) treatment upon autophagic induction (Fig. 4e). Moreover, increased colocalization of LDs with LC3 puncta was observed in PA-treated AML12 cells after Ad-378 infection upon starvation (Fig. 4f). Meanwhile, increased colocalization of LDs with lysosome-associated membrane protein-2 (LAMP2) could be observed by using either BODIPY or Lipidtox, another lipophilic dye for imaging LDs, in AML12 cells overexpressing miR-378 (Fig. 4g; Supplementary Fig. S4g). Collectively, the above results suggest that hepatic miR-378 can reduce the intracellular lipid storage by promoting lysosome-mediated autophagic degradation of LDs in a cell-autonomous manner.

**Figure 5:**
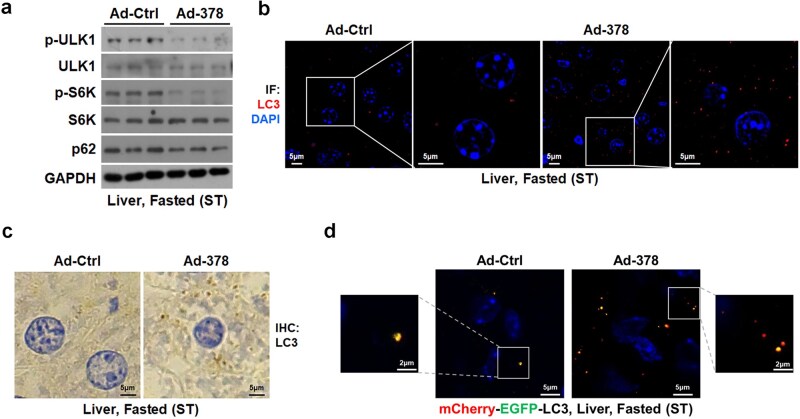
Hepatic miR-378 overexpression promotes autophagy after short-term fasting. (a) Western blot analysis of p-ULK1 (S757), p-S6K (T389), and p62 in the livers of mice infected with Ad-378 after short-term fasting. (b and c) Imunofluorescence (b) and immunohistochemistry (c) staining of LC3 in the livers of mice infected with Ad-378 after short-term fasting. (d) Representative images of mCherry-EGFP-LC3 puncta in the livers of mCherry-EGFP-LC3 mice infected with Ad-378 after short-term fasting.

Subsequently, we investigated the role of miR-378 in regulating hepatic autophagy under short-term fasting, when BA action may not be considerable. After short-term fasting, overexpression of miR-378 in the livers of mice by Ad-378 infection decreased p62 protein accumulation and mTORC1 signaling, as evident from decreased phosphorylation levels of ULK1 and the ribosomal protein S6 kinase (S6K) (Fig. 5a). Accordingly, the number of LC3 puncta was increased after hepatic miR-378 overexpression in these mice (Fig. 5b). Similar results could be obtained from immunohistochemi­stry analysis of LC3 (Fig. 5c). Moreover, Ad-378 infection increased the number of red LC3 puncta in the livers of AFR mice upon short-term fasting, suggesting an increased autophagic flux after miR-378 overexpression (Fig. 5d). Our finding that the pro-autophagic effect of hepatic miR-378 overexpression was accompanied by decreased hepatic TG levels after short-term fasting ­(Supplementary  Fig. S5a) suggested that autophagy enhanced by miR-378 overexpression might contribute to the reduced hepatic lipid accumulation observed. Since miR-378 overexpression did not alter hepatic acetyl-CoA levels, ACOX1 expression, or the mRNA levels of FXR target genes, and circulating BA levels also remained unchanged in short-term fasted mice (Supplementary Fig. S5b–e), we speculated that the inhibitory effect of miR-378 on hepatic autophagy might be outweighed by its pro-autophagic effect, due to the absence of activation in the FXR/ACOX1/acetyl-CoA axis during short-term fasting.

**Figure 6:**
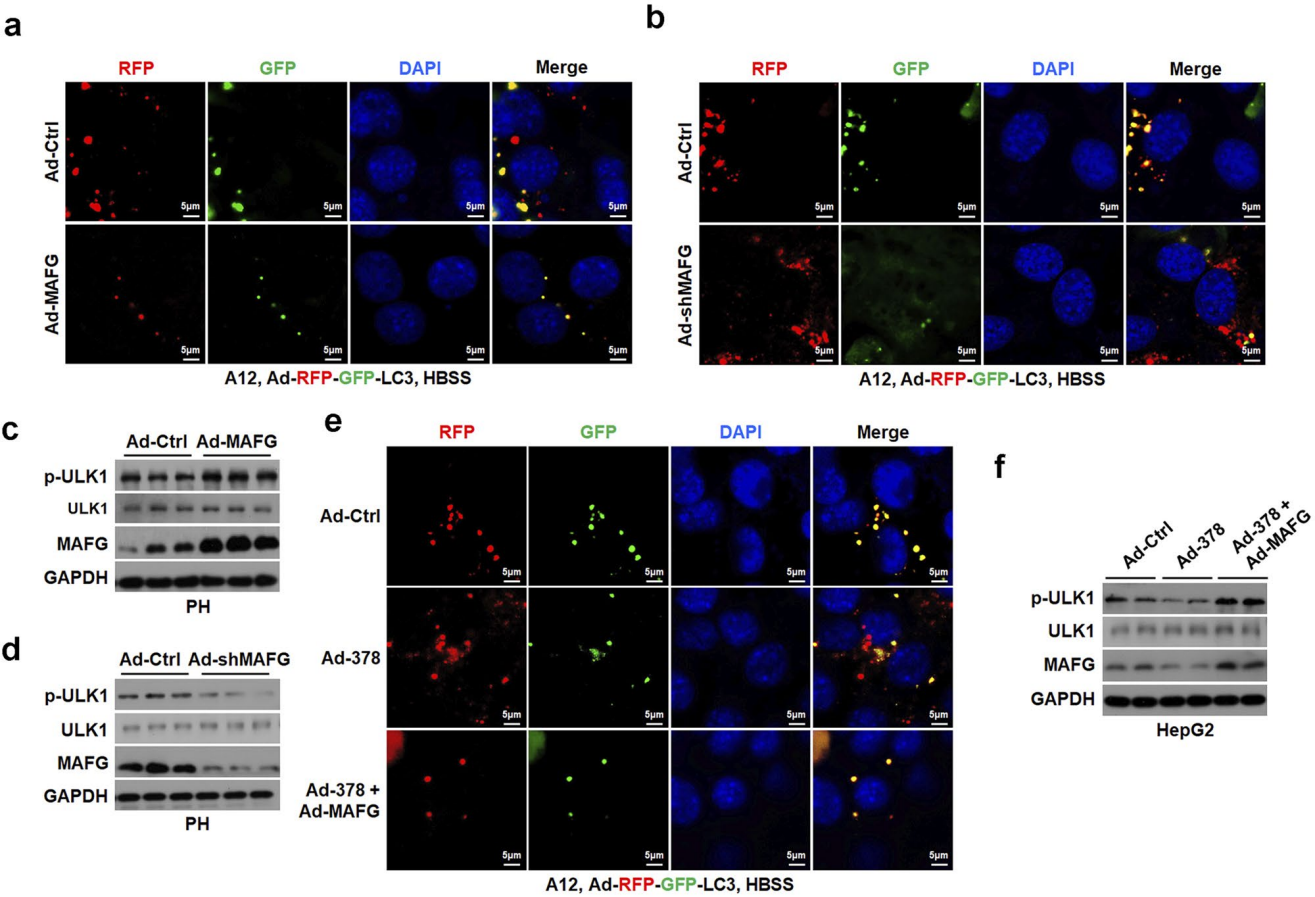
MAFG mediates the promotion of autophagy by hepatic miR-378. (a) AML12 cells co-infected with Ad-MAFG and the LC3 autophagy reporter Ad-RFP-GFP-LC3. (b) AML12 cells co-infected with Ad-sh*MAFG* and the LC3 autophagy reporter Ad-RFP-GFP-LC3. (c) Western blot analysis of p-ULK1 and MAFG in PHs infected with Ad-MAFG. (d) Western blot analysis of p-ULK1 and MAFG in PHs infected with Ad-sh*MAFG*. (e) AML12 cells co-infected with Ad-378 alone or with Ad-378 and Ad-MAFG, together with the Ad-RFP-GFP-LC3 reporter. (f) Western blot analysis of p-ULK1 and MAFG in HepG2 cells infected with Ad-378 and Ad-MAFG as indicated.

Given that the pathways and BA composition regulated by hepatic miR-378 and its target gene *MAFG* were extremely similar (Supplementary Figs. S3b and S6a) [26], we then hypothesized that MAFG is a primary target gene mediating the physiological effects of miR-378 in the liver. To test whether MAFG is involved in the regulation of autophagy by miR-378, we explored the effect of MAFG on hepatic autophagy. Interestingly, overexpression of *MAFG* by adenoviral *MAFG* (Ad-*MAFG*) resulted in a decrease in red fluorescent protein (RFP) signal in AML12 cells co-infected with Ad-RFP-GFP-LC3, suggesting that MAFG is a negative regulator of autophagic flux in hepatocytes (Fig. 6a). On the other hand, inhibition of MAFG by adenoviral *MAFG* shRNA (Ad-sh*MAFG*) led to an increase in RFP signal in AML12 hepatocytes expressing RFP-GFP-LC3 reporter gene (Fig. 6b). Accordingly, Ad-*MAFG* infection increased the phosphorylation of ULK1, while infection with Ad-sh*MAFG* decreased it in cultured hepatocytes (Fig. 6c and d; Supplementary Fig. S6b). Thus, we speculated that MAFG suppresses autophagy by enhancing mTORC1 signaling in hepatocytes. As overexpression of *MAFG* could attenuate the effect of Ad-378 on either autophagic flux or p-ULK1 levels in cultured hepatocytes (Fig. 6e and f), we hypothesized that MAFG is able to mediate the cell-autonomous pro-autophagic effect of miR-378 in hepatocytes.

As the downregulated MAFG protein expression was consistently accompanied with hepatic miR-378 overexpression either due to Ad-378 infection under both short-term and long-term fasting conditions or upon HFD feeding (Supplementary Fig. S6c), we speculated that miR-378-mediated repression of MAFG might be metabolic context independent. Since manipulation of either MAFG expression or CDCA treatment was sufficient to modulate autophagy in PHs from mice under various conditions, including short-term fasting, long-term fasting, and HFD feeding ­(Supplementary  Fig. S6d and e), we collectively speculated that the role of MAFG or FXR in regulating autophagy might be cell-autonomous and independent of the metabolic context.

Taken together, our results support the hypothesis that miR-378 in the liver exerts dichotomous effects on hepatic autophagy, with its net outcome being ultimately determined by the metabolic context (Supplementary Fig. S6f). Under short-term fasting conditions, where BA signaling is minimal and the FXR/ACOX1/acetyl-CoA axis remains inactive, the pro-autophagic effect of miR-378, which stems from its suppression of MAFG, dominates. Conversely, after prolonged fasting or HFD feeding, the downregulation of MAFG by miR-378 leads to substantial BA accumulation. This, in turn, activates the FXR/ACOX1/acetyl-CoA axis, which exerts a potent inhibitory effect on autophagy that outweighs the direct pro-autophagic effect of MAFG suppression (Supplementary  Fig. S6g). Thus, our study unveils previously unrecognized physiological and pathological functions of BAs in regulating hepatic autophagy and establishes both hepatic miR-378 and circulating BAs as bona fide regulators of hepatic autophagy and lipid catabolism during fasting.

### Hepatic zonation of miR-378 levels and autophagy in different nutritional states

**Figure 7:**
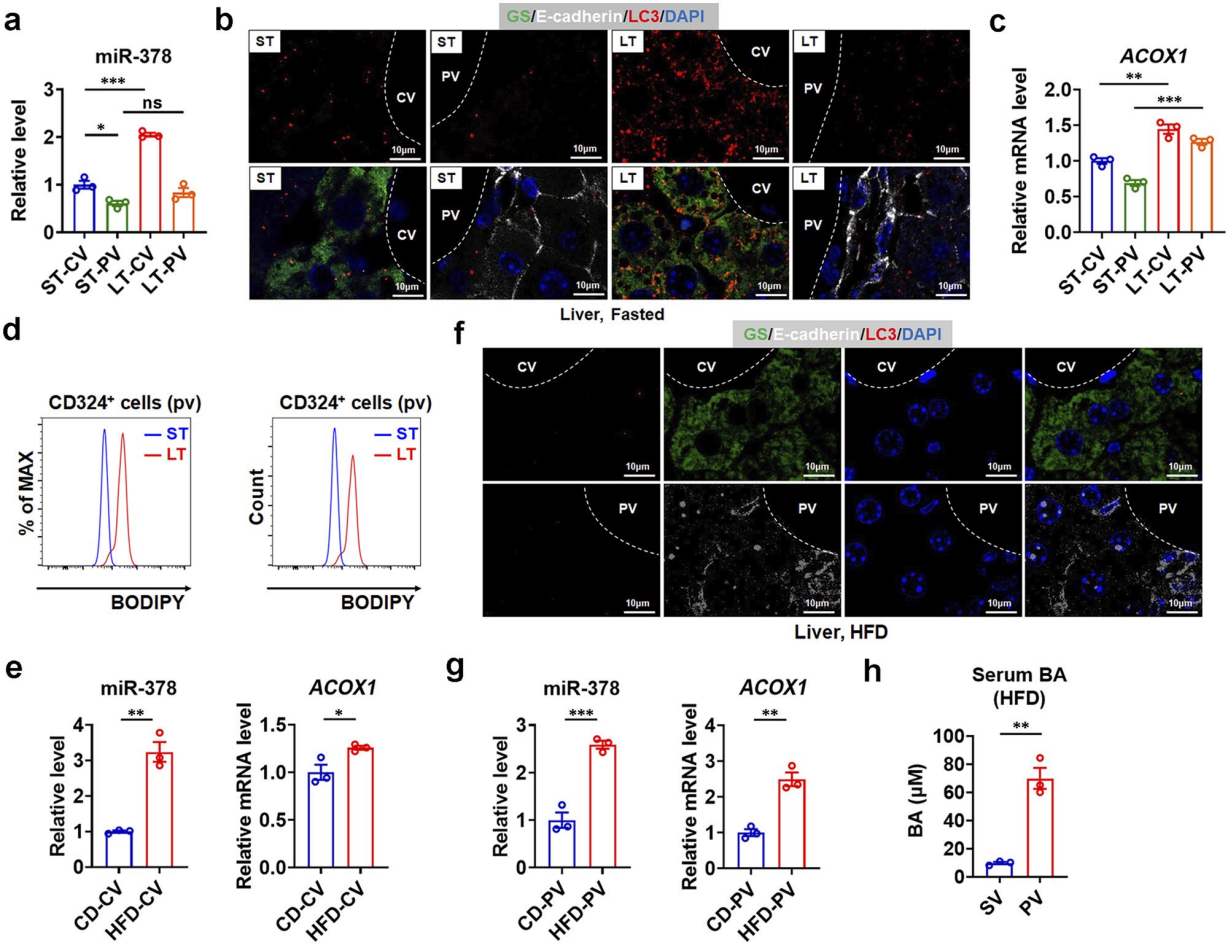
Hepatic zonation of miR-378 levels and autophagy under various nutritional conditions. (a) Relative miR-378 levels in CV and PV hepatocytes of fasted mice (*n *= 3). (b) Immunofluorescent staining of LC3, E-cadherin, and GS in the livers of fasted mice. (c) Relative mRNA levels of *ACOX1* in CV and PV hepatocytes of fasted mice (*n *= 3). (d) Representative flow cytometry histogram of BODIPY fluorescence in CD324^+^ PV cells from fasted mice. (e) Relative expression of miR-378 and ACOX1 in CV hepatocytes of HFD-fed mice (*n *= 3). (f) Immunofluorescent staining of LC3, E-cadherin, and GS in the livers of HFD-fed mice. (g) Relative expression of miR-378 and ACOX1 in PV hepatocytes of HFD-fed mice (*n *= 3). (h) The levels of systemic venous (SV) BAs and portal venous (PV) BAs in HFD-fed mice (*n *= 3). ST and LT stand for short-term and long-term, respectively. Means ± SEM are shown. *P* values were calculated by two-tailed unpaired Student’s *t*-test. ns, not significant. ^*^P < 0.05; ^**^P < 0.01; ^***^P < 0.001.

Finally, as autophagic zonation has been reported very recently [27], we then tested whether miR-378 exhibits a zonated expression pattern, thereby being critically involved in the spatiotemporal regulation of hepatic autophagy under different nutritional status. We isolated the pericentral hepatocytes around central vein (CV) (CV hepatocytes) and periportal hepatocytes around PV (PV hepatocytes), respectively, by using flow cytometry, and confirmed the expression of marker genes (Supplementary Fig. S7a–c and e). Interestingly, after short-term (ST) fasting, miR-378 was expressed mainly in pericentral hepatocytes, accompanied by a zonated autophagy with a similar bias, as reflected by a pericentral enrichment of red LC3 puncta (ST-CV versus ST-PV) (Fig. 7a and b). These results suggest that the zonal pattern of hepatic autophagy after short-term fasting might be attributed, at least in part, to the zonated expression of miR-378 and its cell-autonomous pro-autophagic action.

Autophagy was further enhanced in pericentral hepatocytes after long-term (LT) fasting, accompanied by further elevation of miR-378 levels (ST-CV versus LT-CV) (Fig. 7a and b). As the levels of pericentral ACOX1 were increased after long-term fasting (ST-CV versus LT-CV) (Fig. 7c), likely due to the elevation of BA levels (Fig. 2n), we speculated that the greatly enhanced pericentral autophagy after long-term fasting might be principally attributed to the cell-autonomous pro-autophagic effect of miR-378 rather than the increase in anti-autophagic ACOX1 expression, although the action of circulating BAs became important after long-term fasting (Fig. 2n).

Notably, the degree of autophagy enhancement around PV (ST-PV versus LT-PV) was not as significant as that around CV (ST-CV versus LT-CV) (Fig. 7b). As only the mRNA levels of periportal *ACOX1* showed an increase after long-term fasting, not the levels of periportal miR-378 (Fig. 7a and c), and the uptake of portal venous BAs is predominantly in the PV zones [28], we speculated that the hindered increase in periportal autophagy might be mainly ascribed to the anti-autophagic effect of BAs, mediated through FXR-induced upre­gulation of ACOX1 after long-term fasting when the action of circulating BAs became considerable (Fig. 2n). Nevertheless, we could not overlook the impact from non-increasable expression of periportal miR-378 (Fig. 7a). Similarly, taking into account the importance of BAs returning to the liver, we also speculated that the hindered increase in periportal autophagy might contribute to the lipid accumulation in the periportal hepatocytes, as evident from stronger staining of LDs, after long-term fasting (Fig. 7d).

Interestingly, although HFD feeding resulted in an increase in miR-378 expression in pericentral hepatocytes (Fig. 7e), the increased miR-378 expression did not result in a marked increase in pericentral autophagy in these mice as it did in mice fed a chow diet after long-term fasting (LT-CV versus HFD-CV) (Fig. 7b and f; Supplementary Fig. S7d), indicating a miR-378 resistance status in the pericentral hepatocytes of HFD-fed mice. Since HFD feeding also resulted in an increase in pericentral ACOX1 expression (Fig. 7e), we speculated that after HFD feeding when BA levels were elevated and their action became considerable (Fig. 1c), the increased expression of ACOX1 might serve as a mechanism for the development of miR-378 resistance, impairing the ability of miR-378 in pericentral hepatocytes to promote autophagy. Notably, we observed an elevation of both miR-378 and *ACOX1* mRNA levels in the periportal hepatocytes of HFD-fed mice (Fig. 7g). The levels of portal venous BAs were higher than those of systemic venous BAs in HFD-fed mice (Fig. 7h). As LC3 puncta were almost undetectable in the periportal hepatocytes of HFD-fed mice (Fig. 7f), we speculated that the absence of autophagy might be primarily attributed to the anti-autophagic effect of BAs, mediated through FXR-induced upregulation of ACOX1 upon HFD feeding when the action of BAs became considerable (Fig. 1c). Based on our findings here, we also speculated that the impaired autophagy in both pericentral and periportal hepatocytes contributes to the abnormal lipid accumulation in the liver after HFD feeding (Supplementary Fig. S7f), regardless of the underlying differences in mechanisms.

## Discussion

Autophagy plays a vital role in modulating lipid homeostasis through mediating intracellular lipid breakdown. Notably, defective autophagy is closely associated with MASLD; however, the underlying mechanisms are uncertain. Growing evidence indicates that MASLD is associated with alterations in BAs, while BAs have been shown to regulate autophagy. In this study, we found that patients with hepatic steatosis exhibited elevated circulating BA levels. Moreover, elevated levels of circulating BAs were independently associated with an increased risk of hepatic steatosis. Consistently, HFD-fed mice showed elevated circulating and hepatic BA levels along with impaired autophagy. Moreover, we showed that inhibition of FXR by UDCA could restore defective autophagy and ameliorate liver steatosis in HFD-fed mice. We speculate that elevated BA levels may promote hepatic steatosis by activating FXR to suppress autophagy after overfeeding. Thus, our study not only unco­vers the pathological roles of elevated BA levels and sustained activation of hepatic FXR in MASLD but also provides the foundation for using FXR antagonists to treat MASLD with autophagy dysfunction.

It has been proposed that FXR acts to repress hepatic autophagy by downregulating the expression of autophagy components at the transcriptional level [18, 19]. However, our data indicate that FXR activation by GW4064 did not significantly affect the transcription of key autophagy-related genes in PHs, suggesting that FXR may regulate autophagy via transcription-independent mechanisms. Although previous reports indicate that the regulation of autopha­gy by FXR is likely mTOR-independent, which largely relies on the data of phosphorylated S6 (Ser240/244) rather than phosphorylated ULK1 (Ser757) (more related to autophagy), the association of increased ULK1 phosphorylation with FXR activation has been noticed [18, 29]. Acetyl-CoA, a crucial metabolic intermediate, has recently been recognized as an important regulator of autophagy through activation of mTORC1 signaling pathway. Our data suggest that FXR activation leads to an increase in acetyl-CoA production by elevating the ACOX1 expression, and subsequently suppresses autophagy by enhancing mTORC1−ULK1 signaling. Thus, based on our data, we believe that we discovered a previously undescribed mechanism underlying the regulation of hepatic autophagy by FXR, which involves the acetyl-CoA-regulated mTORC1−ULK1 signaling.

Prolonged fasting can lead to physiological hepatic steatosis. We found that circulating BA levels were increased after prolonged fasting. As BA serves as a key component of bile, which is produced by the liver and secreted into the duodenum to aid in the digestion and absorption of fats after a meal, its receptor FXR has been natu­rally considered as a fed-state sensor [30, 31]. For the same reason, the function of either BA or FXR is rarely investigated during fasting. Although the inhibitory effect on hepatic autophagy by activation of FXR has been noticed even in fasted mice, in previous studies, the regulation of autophagy by hepatic FXR was normally mentioned or discussed in the fed state or under postprandial conditions [18, 19, 32]. According to our knowledge, the physiological roles of both BA and FXR during prolonged fasting may be underestimated as compared to another metabolic nuclear receptor, fatty acid-activated peroxisome proliferator-activated receptor-α (PPARα). In this study, our data suggest that BA and its receptor FXR play important roles in autophagy and lipid homeostasis after prolonged fasting, which has not been previously appreciated.

We have previously established that hepatic miR-378 promotes BA synthesis by suppressing MAFG, a transcriptional repressor of key enzymes in both classical and alternative BA synthesis pathways [23]. In this study, we found that the elevated total BA levels after prolonged fasting due to hepatic overexpression of miR-378 was accompanied by increased expression of FXR target genes (Supplementary Fig. S3c). Given that it is well-accepted that the postprandial rise in BAs inhibits their own synthesis through activation of the nuclear receptor FXR, our findings suggest that the elevation of total BAs driven by miR-378 overexpression after prolonged fasting may be sufficient to activate FXR, thereby suppressing autophagy and promoting hepatic lipid accumulation in the liver. In addition to the rise in total BAs, we noted an increase in agonistic species, attributable to the shift towards 12α-hydroxylated BAs (Supplementary Fig. S3b), probably due to the action of MAFG on cytochrome P450 family 8 subfamily B member 1 (CYP8B1), which might further facilitate FXR activation. However, any potential synergy from this shift remains to be validated.

In this study, we demonstrate that hepatic miR-378 is upregulated under steatotic conditions induced by prolonged fasting or an HFD. We further establish that miR-378 exerts dual and opposing effects on hepatic autophagy through two distinct mechanisms: a cell-autonomous pro-autophagic effect via mTORC1 suppression, and a systemic anti-autophagic effect mediated by MAFG/BA/FXR/ACOX1 axis, which elevates acetyl-CoA levels to stimulate mTORC1. The net autophagic outcome is determined by metabolic context. During short-term fasting, the cell-autonomous effect dominates due to limited BA elevation (Supplementary Fig. S6g). In contrast, prolonged fasting or HFD feeding enables the MAFG-mediated rise in BAs to activate the FXR pathway, whose systemic anti-autophagic signal then overrides the cell-autonomous effect (Supplementary Fig. S6g). Therefore, the shift in autophagic outcome stems not from altered functions of MAFG or FXR, but from the delayed systemic manifestation of MAFG inhibition on BA levels, which subsequently engages the potent BA/FXR inhibitory axis.

The mitochondrial acetyl-CoA, derived from glycolysis or β-oxidation, is normally committed to TCA cycle for energy production and is not directly available to the cytosol. The cytosolic acetyl-CoA pool can be generated from very-long-chain fatty acyl-CoAs via ACOX1, from mitochondria-derived citrate via ACLY, or from acetate via ACSS2. In this study, given that either FXR activation by GW4064 *in vitro* or prolonged fasting specifically upregulated ACOX1 without altering ACLY or ACSS2 expression (Supplementary Fig. S2e and j), we focused our investigation on the contribution of ACOX1 under these metabolic conditions. Our further study suggests that ACOX1 can serve as a key contributor to increased acetyl-CoA production and suppressed autophagy in the liver after prolonged fasting. Our current data do not allow us to rule out the involvement of other contributing mechanisms. It is unclear whether the starvation-induced shift towards β-oxidation over glycolysis sufficiently increases mitochondrial acetyl-CoA to drive the export of citrate, and whether this citrate flux can override reduced ACLY-mediated generation to ultimately raise cytosolic acetyl-CoA pools. Further analysis, particularly using detailed metabolic flux measurements, will be needed to provide direct evidence to validate the proposed model, test for alternative mechanisms, and assess their relative contributions.

Taken together, our results establish novel physiological and pathological functions of BAs in modulating autophagy and lipid metabolism under conditions of prolonged fasting and MASLD. Mechanistically, circulating BAs promote acetyl-CoA production via hepatic FXR-mediated ACOX1 expression, thereby suppressing autophagy through increasing mTOR signaling.

### Limitations of the study

Although this study reveals a pivotal role of the BA/FXR/ACOX1/acetyl-CoA axis in suppressing hepatic autophagy, several limitations remain. First, the role of alternative acetyl-CoA-generating pathways, such as those mediated by ACLY and ACSS2, was not fully explored. Therefore, the precise quantitative contribution of ACOX1-derived acetyl-CoA relative to other sources remains unclear. Future studies employing isotope-tracing and detailed metabolic flux analysis *in vivo* will be essential to comprehensively understand the regulation of autophagy and lipid metabolism under different nutritional and pathological conditions. Second, the intriguing zonation of miR-378 and autophagy, while suggestive of a spatially resolved mechanism, remains correlative. The relationship between pericentral miR-378 expression and zonated autophagy requires direct experimental validation. Future studies employing zonation-specific gene manipulation will be able to dissect the cell-autonomous and non-cell-autonomous mechanisms of miR-378 in distinct hepatic zones.

## Materials and methods

### Animal and human studies

All animal experiments were performed in accordance with institutional guidelines for the care and use of animals. Animal protocols were approved by the Ethics Committee of Shanghai Institute of Nutrition and Health, Chinese Academy of Sciences Animal Care Committee (SINH-2022-YH-1). Male C57BL/6 mice and transgenic mice on the C57BL/6 background aged approximately 2–3 months were used in this study. Mice were housed under a 12 h light/12 h dark cycle in a specific pathogen-free animal facility. AFR mice (mCherry-EGFP-LC3; Shanghai Model Organisms Center, Inc.) were crossed with Alb-Cre mice. Adenoviruses were delivered via tail-vein injection to overexpress hepatic miR-378. Each mouse received Ad-378 (5 × 10^8^ plaque-forming units [PFU]) or Ad-Ctrl (5 × 10^8^ PFU) for 2 weeks. For short-term fasting experiments, mice were kept in new cages without food for 4–6 h before sacrifice. For long-term fasting experiments, mice were kept in new cages without food for 24–36 h before sacrifice. All mice had unlimited access to water. To induce hepatic steatosis, mice aged 6–8 weeks were fed on an HFD containing 60 kcal% fat (D12492, Research Diets) for 12 weeks. For UDCA treatment, following 12 weeks of HFD feeding, mice were maintained on an HFD supplemented with or without 0.5% (w/w) UDCA for an additional 2 months.

Data for 608 human subjects with and without hepatic steatosis were obtained from Zhongshan Hospital, Fudan University. The clinical characteristics are shown in Supplementary Table S1. The diagnosis of hepatic steatosis was based on abdominal ultrasound examination. The study was approved by the Ethics Committee of Zhongshan Hospital, Fudan University. Informed consent was obtained from all participants.

### Cell culture and treatment

AML12 cells were cultured in DMEM/F12 supplemented with 10% fetal bovine serum (FBS), 1% insulin-transferrin-selenium (ITS), 1% penicillin/streptomycin and 40 ng/mL dexamethasone. Primary mouse hepatocytes and HepG2 cells were cultured in DMEM supplemented with 10% FBS and 1% penicillin/streptomycin. Adenoviruses for the overexpression of miR-378 (Ad-378), MAFG (Ad-MAFG), and knockdown of *MAFG* (Ad-sh*MAFG*) were generated as described previously [26]. Cells were infected with the adenoviruses at a multiplicity of infection (MOI) of 10 for 36 h. GMR-miR^TM^ microRNA double-stranded mimics for miR-378 (ACU GGA CUU GGA GUC AGA AGG), an inhibitor for miR-378 (CCU UCU GAC UCC AAG UCC AGU), and scrambled controls were purchased from GenePharma. To investigate the role of miR-378 *in vitro*, cells were transfected with the indicated RNA oligonucleotides using Lipofectamine 2000 (Thermo Fisher) according to the manufacturer’s instructions. AML12 cells were infected with Ad-RFP-GFP-LC3 ­(Hanbio Co. Ltd) for 36 h. AML12 cells stably expressing mRFP-GFP-LC3 were established via lentiviral infection. For HBSS starvation, cells were incubated in HBSS media for 2 h before analysis unless otherwise stated. For lipophagy studies, cells were incubated with 100 μmol/L PA for 24 h, followed by 2-h starvation in HBSS before fixation. To block autophagic flux, cells were treated with either 200 μmol/L CQ or 200 nmol/L Baf-A1 for 2 h before collection. For CDCA treatment, cells were treated with 25 μmol/L CDCA or vehicle for 24 h. For GW4064 treatment, cells were exposed to 2 μmol/L GW4064 or vehicle in complete medium for 24 h unless otherwise indicated. For (-)-hydroxycitric acid (HCA) treatment, cells were treated with 200 μmol/L HCA for 2 h. To mimic the nutritional status during prolonged fasting, PHs were incubated in low-glucose (1 g/L) DMEM supplemented with 50 µmol/L oleic acid (OA), 50 μmol/L PA, 10% FBS, and 1% penicillin/streptomycin for 24 h.

### Immunofluorescence, immunohistochemical, and histological staining

Liver tissues were fixed with 4% paraformaldehyde and embedded in paraffin or snap-frozen in optimal cutting temperature (OCT) compound. For immunofluorescence staining, tissue sections were fixed in 4% paraformaldehyde and deparaffinized with xylene and ethanol, followed by heat-activated antigen retrieval. Slides were blocked with 5% goat serum for 1 h at room temperature and incubated overnight at 4 °C with the following primary antibodies: anti-LC3B (2775S, CST, 1:100), glutamine synthetase (GS, 11037-2-AP, Proteintech, 1:200), p62 (66184-1-Ig, Proteintech, 1:200), and E-cadherin (3195S, CST, 1:200). The next day, slides were washed in PBS and incubated with appropriate fluorescent secondary antibodies (1:1000) for 1 h at room temperature. Finally, nuclei were stained with DAPI. For LD staining, frozen liver sections were incubated with LipidTOX (H34476, Invitrogen). For cell imaging, hepatocytes were fixed in 4% paraformaldehyde, blocked, permeabilized, and incubated with the primary antibodies including anti-LC3B (2775S, CST, 1:100) and anti-LAMP2 (34141, CST, 1:100), followed by incubation with secondary antibodies. For LD staining in cells, cells were incubated with LipidTOX or BODIPY (493/503) (D3861, ­Invitrogen). DAPI was used to stain nuclei. For histological staining, paraffin-embedded sections were stained with hematoxylin and eosin (H&E). For immunohistochemistry, paraffin sections were stained with the primary antibody anti-LC3B (2775S, CST, 1:100).

### Biochemical studies

Liver TG content was measured according to the manufacturer’s instructions (290-63701, Wako). Briefly, approximately 50 mg of liver tissue was homogenized in 300 μL of isopropyl alcohol and centrifuged. After centrifugation, liver TGs were assessed using the LabAssay Triglyceride measurement kit. Hepatic acetyl-CoA levels in the liver were measured using an acetyl-CoA assay kit (MAK039, Sigma) according to the manufacturer’s instructions. Total BA levels in the serum and the liver were analyzed using the Total Bile Acid Assay Kit (E003-2-1, Jian-Cheng). The individual BA species in the serum were quantified using an ultra-high performance liquid chromatography (UHPLC) system coupled with an Orbitrap Exploris 120 mass spectrometer (Thermo Fisher Scientific) (Supplementary Table S2).

### Analyses of mRNA and protein levels

Total RNA was extracted from tissues or cells using TRIzol reagent (15596018, Invitrogen) according to the manufacturer’s instructions. Reverse transcription was subsequently performed with the RT Reagent Kit (RR037B, Takara). For real-time quantitative PCR (RT-qPCR) analysis, reactions were conducted using SYBR Green PCR Master Mix (11203ES08, YEASEN) on a QuantStudio 6 Flex Real-Time PCR System (ThermoFisher). Relative gene expression levels were calculated using the comparative ΔΔCt method. Cycle threshold (Ct) values were normalized to 18S rRNA as the internal reference. Primer sequences are provided in Supplementary Table S3. RNA-seq libraries were constructed using the Illumina TruSeq Stranded mRNA Library Preparation Kit (Illumina). Samples were pooled for deep sequencing on NovaSeq X Plus platform. Differential gene expression analysis was performed using the DEGSeq. Data analysis was carried out via the Majorbio Cloud Platform (majorbio.com). The data have been deposited in the NODE database (OEP00006649).

For western blot analysis, proteins were extracted from cells or tissues using radio-immuno precipitation assay (RIPA) lysis buffer supplemented with protease and phosphatase inhibitors, and quantified using the Bradford assay (23236, ThermoFisher). Lysates were separated by SDS-PAGE and transferred onto polyvinylidene fluoride (PVDF) membranes. Membranes were blocked with 5% BSA and incubated overnight at 4 °C with primary antibodies, including: p62 (610833, BD Biosciences), p-S6K(T389) (9234, CST), S6K (34475, CST), β-Actin (A5316, Sigma), LC3B (2775S, CST), Hsp90 (4874S, CST), p-ULK1(S757) (6888S, CST), ULK1 (8054S, CST), MAFG (NBP2-15019, Novus Biologicals), ACOX1 (10957-1-AP, Proteintech), and GAPDH (10494-1-AP, Proteintech). Chemiluminescent signals were detected using BeyoECL Plus (P0018S, Beyotime).

### ChIP assay

ChIP assays were conducted using the EZ Magna ChIP G kit (17-409, Merck/Millipore) following the manufacturer’s recommended protocol. Primary mouse hepatocytes were incubated with 2 μmol/L GW4064 or vehicle for 24 h. Chromatin lysates were immunoprecipitated with an anti-FXR antibody (sc-13063, Santa Cruz) or anti-mouse IgG (12-371B, Millipore) as a negative control. The PCR products were analyzed by electrophoresis on a 2% agarose gel. Primer sequences for ChIP assay are provided in Supplementary Table S3.

### Primary mouse hepatocyte isolation and fluorescence-activated cell sorting (FACS) analysis

The isolation of PHs was performed as previously described [33], with minor modifications. In brief, after mice were anesthetized, the livers were perfused with perfusion buffer (PBS with 1 mmol/L EGTA), followed by digestion buffer (0.05% collagenase type I). Isolated hepatocytes were filtered through a 70-µm cell strainer. The resulting cell suspension was centrifuged at 50 *g* for 2 min at 4 °C. To remove dead hepatocytes, the pellet was resuspended with 12.5 mL DMEM and added to 11.25 mL Percoll mixed with 1.25 mL 10× HBSS. Cells were then centrifuged at 1000 *g* for 10 min. The centrifuged cells were collected and counted. For FACS analysis, hepatocytes were centrifuged at 50 *g* for 2 min at 4 °C, resuspended in FACS buffer (2 mmol/L EDTA and 0.5% BSA in 1× PBS), and filtered through a 70-µm cell strainer. Dead cells were stained with Live/Dead Violet Viability Kit (Invitrogen) and excluded during analysis. An anti-mouse CD16/32 antibody (Invitrogen) was used to block non-specific binding. To sort pericentral and periportal hepatocytes, cells were stained with APC anti-mouse CD73 (1:100, BioLegend) and PE anti-mouse/human CD324 E-cadherin (1:100, BioLegend). The protocol was developed as described before [34]. Flow ­cytometry data were acquired using a CytoFLEX S flow cytometer ­(Beckman). FACS was performed on MoFlo Astrios EQ sorter.

### Targeted metabolomics analysis

Targeted metabolomics analysis was performed using a UHPLC system coupled to a triple quadrupole mass spectrometer, with metabolites detected in both positive and negative electrospray ionization modes. Widely targeted metabolites were quantified in multiple-reaction monitoring (MRM) mode, with all transitions from large-scale metabolites (QMT1000 Kit, Shanghai BioProfile) detected under optimized declustering potential and collision energy. The original MRM data were extracted using MultiQuant 3.0.2 software, and the peak area of each metabolite was obtained for quantification across all samples. The data have been deposited in the NODE database (OEZ00021653).

### Statistical analysis

Randomization and blinding strategy were used whenever possible. All experiments were performed at least twice, and representative data are shown. Data were expressed as means ± SEM. GraphPad Prism and two-tailed unpaired Student’s *t-*test were applied. Logistic regression models were used to estimate the values of OR with 95% CI. Zen 3.8 (Blue edition) software was used for acquisition of images from confocal microscope. FlowJo was used for FACS analysis. A *P*-value of less than 0.05 was considered significant.

## Supplementary Material

loaf038_Supplementary_Data

## Data Availability

H.Y. is the guarantor of this work and, as such, had full access to all the data in the study and takes responsibility for the integrity of the data and the accuracy of the data analysis. The authors confirm that all the data supporting the findings of this study are available within the supplementary material and corresponding authors.
