# Supplementary material for: Aberrant miR-378 expression promotes hepatic lipid accumulation via hijacking the bile acid-regulated autophagy
Source: Life Metab. 2025 Nov 10;5(1):loaf038. doi: 10.1093/lifemeta/loaf038 (PMC12990297; doi:10.1093/lifemeta/loaf038)
Supplement: loaf038_Supplementary_Data [file loaf038_supplementary_data.pdf]

Supplementary Information

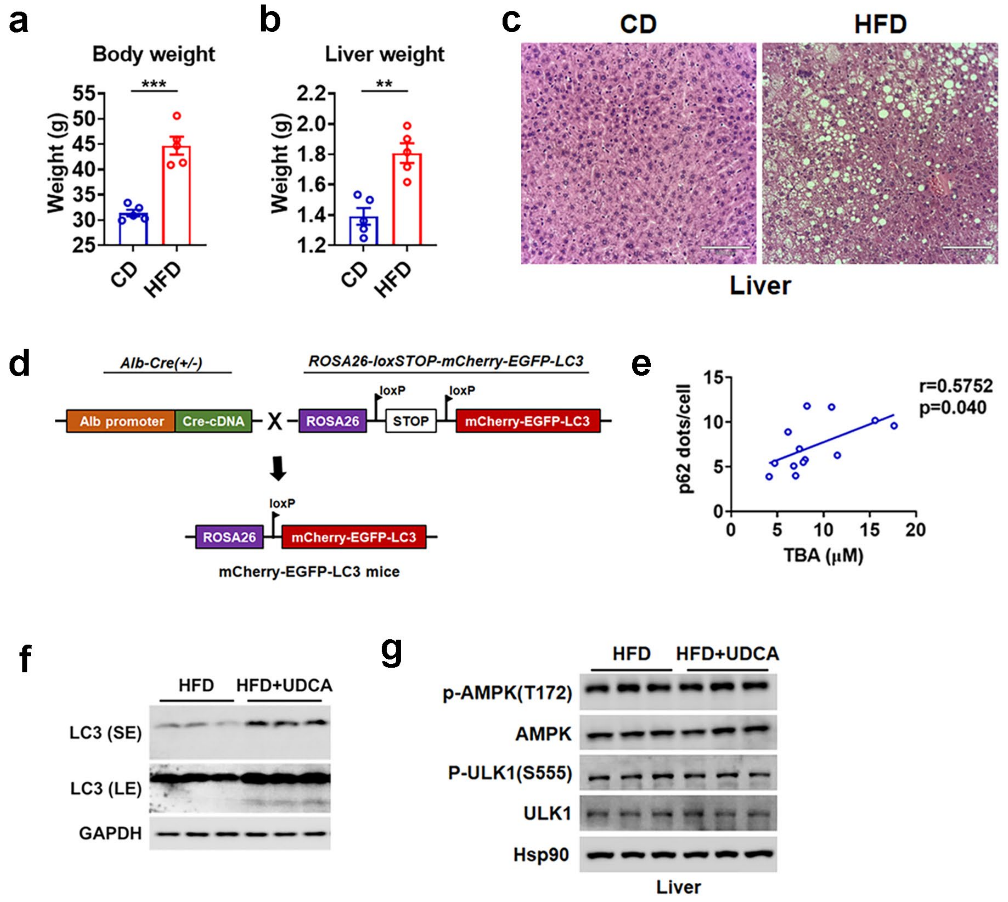

Supplementary Figure S1 related to Figure 1. (a and b) Body weight and liver weight of HFD-fed mice (n = 5). (c) Representative images of hematoxylin-eosin (H&E) staining of liver sections harvested from HFD-fed mice. (d) Breeding scheme for generating autophagic flux reporter (AFR) mice expressing Cre recombinase under the control of albumin promoter (Alb-Cre). (e) Association analysis of serum BA levels and hepatic p62 levels in HFD-fed mice (n = 13). (f) Western blot analysis of LC3 in the livers of HFD-fed mice treated with UDCA. SE, short exposure; LE, long exposure. (g) Western blot analysis of p-AMPK (T172) and p-ULK1 (S555) in the livers of HFD-fed mice treated with UDCA. Means ± SEM are shown. P values were calculated by two-tailed unpaired Student's t-test. $^{**}P < 0.01$; $^{***}P < 0.001$.

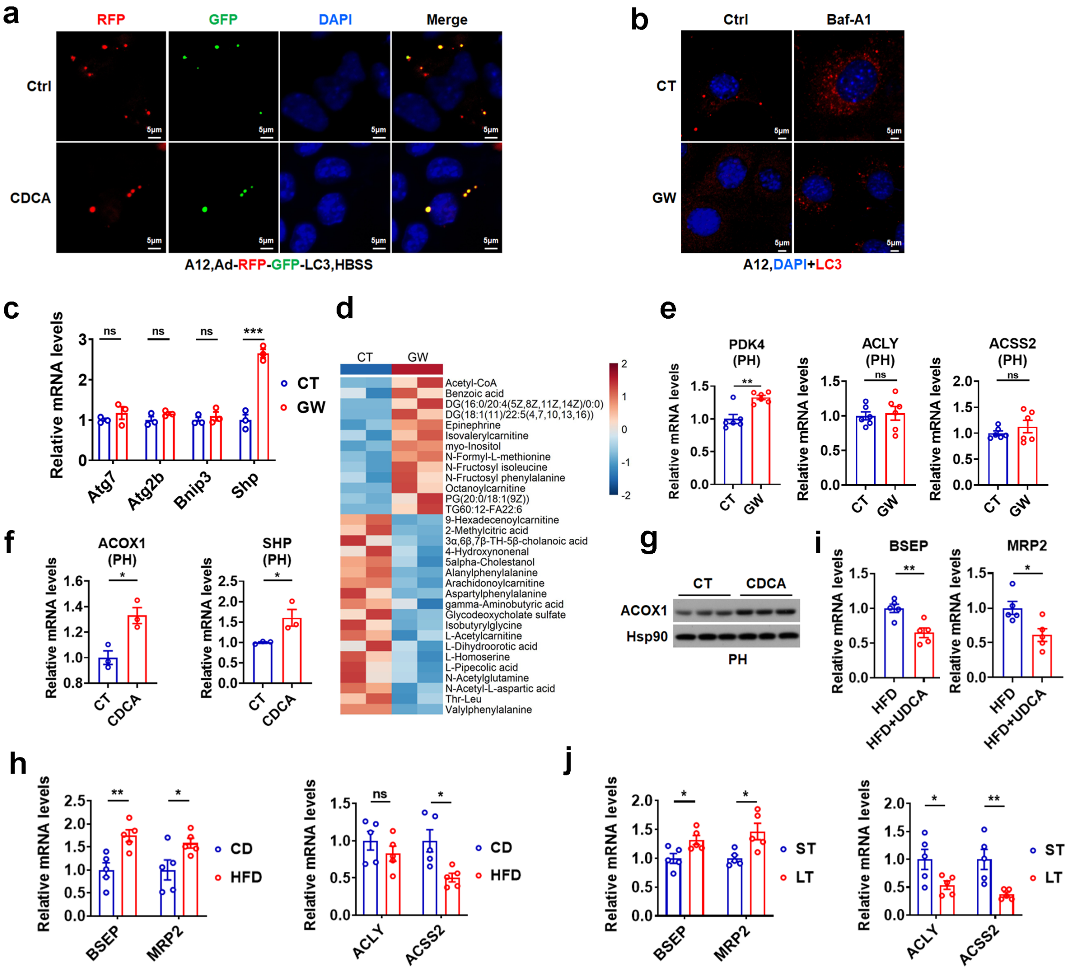

Supplementary Figure S2 related to Figure 2. (a) AML12 cells infected with the LC3 autophagy reporter RFP-GFP-LC3 were treated with CDCA, followed by 2 h of HBSS starvation. (b) Immunofluorescent staining of LC3 in AML12 cells treated with GW4064 in the absence or presence of Baf-A1. (c) Relative mRNA levels of Atg7, Atg2b, Bnip3, and Shp in PHs treated with GW4064 (n = 3). (d) Heat map of metabolomic profiles in primary hepatocytes from vehicle control (CT) and GW4064 (GW)-treated groups, showing metabolites with fold change > 2. (e) Relative mRNA levels of PDK4, ACLY, and ACSS2 in PHs treated with GW4064 (n = 6). (f and g) Relative mRNA levels of ACOX1 and SHP (f) and western blot analysis of ACOX1 (g) in PHs treated with 25 μmol/L CDCA (n = 3). (h) Relative mRNA levels of FXR target genes (*BSEP* and *MRP2*), ACLY, and ACSS2 in the livers of HFD-fed mice (n = 5). (i) Relative mRNA levels of FXR target genes (*BSEP* and *MRP2*) in the livers of HFD-fed mice treated with UDCA (n = 5). (j) Relative mRNA levels of FXR target genes (*BSEP* and *MRP2*), ACLY, and ACSS2 in the livers of short- and long-term fasted mice (n = 5). Means ± SEM are shown. P values were calculated by two-tailed unpaired Student's t-test. $^{*}P < 0.05$; $^{**}P < 0.01$; $^{***}P < 0.001$. ns, not significant.

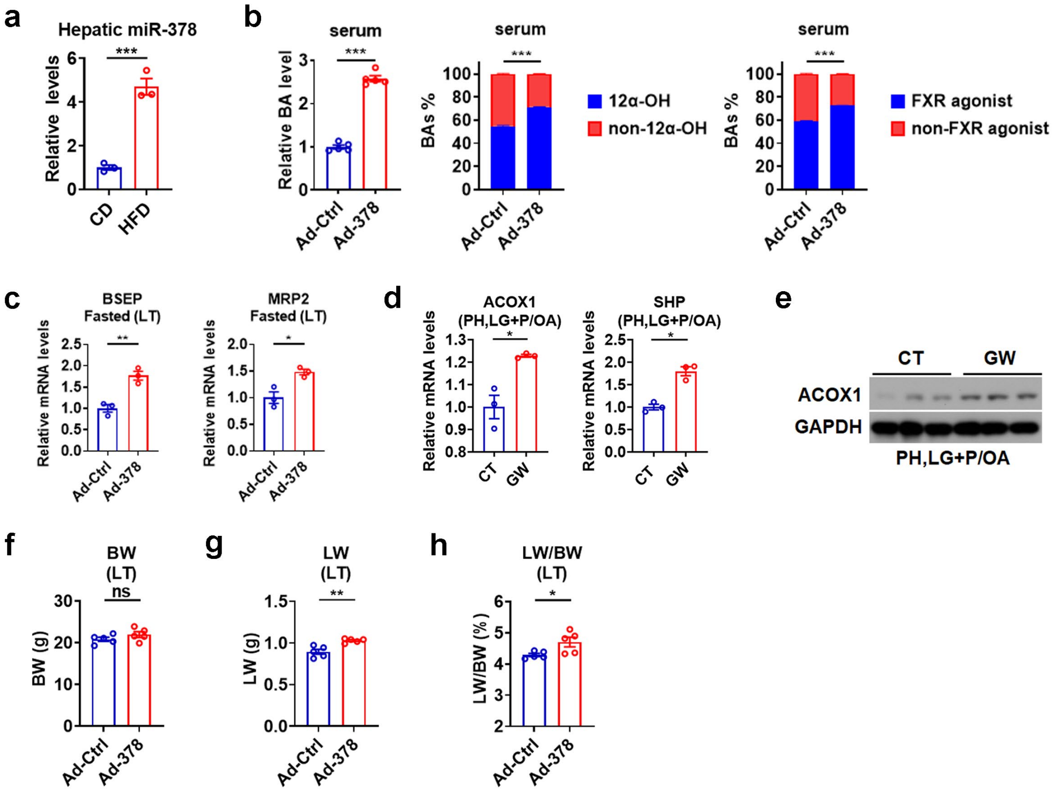

Supplementary Figure S3 related to Figure 3. (a) Relative miR-378 levels in the livers of HFD-fed mice (*n* = 3). (b) Relative total BA levels and percentages of 12α-OH BAs (including CA, DCA, T(G)-CA, and T-DCA) and FXR-agonistic BAs (including CA, CDCA, DCA, T(G)-CA, T(G)CDCA, and T-DCA) in the sera of Ad-378-infected mice after long-term fasting, analyzed by LC-MS/MS (*n* = 5). (c) Relative mRNA levels of FXR target genes (*BSEP* and *MRP2*) in the livers of Ad-378-infected mice after long-term fasting (*n* = 3). (d and e) Relative mRNA levels of ACOX1 and SHP (d) and western blot analysis of ACOX1 (e) in PHs pretreated with palmitic acid/oleic acid (P/OA) for 24 h in the presence or absence of GW4064 in low-glucose medium (*n* = 3). (f and g) Body weight (BW) and liver weight (LW) of mice infected with Ad-378 after long-term fasting (*n* = 5). (h) The ratio of liver weight to body weight (LW/BW) in mice infected with Ad-378 after long-term fasting (*n* = 5). LT stands for long-term. Means ± SEM are shown. *P* values were calculated by two-tailed unpaired Student's *t*-test. $^{*}P < 0.05$; $^{**}P < 0.01$; $^{***}P < 0.001$. ns, not significant.

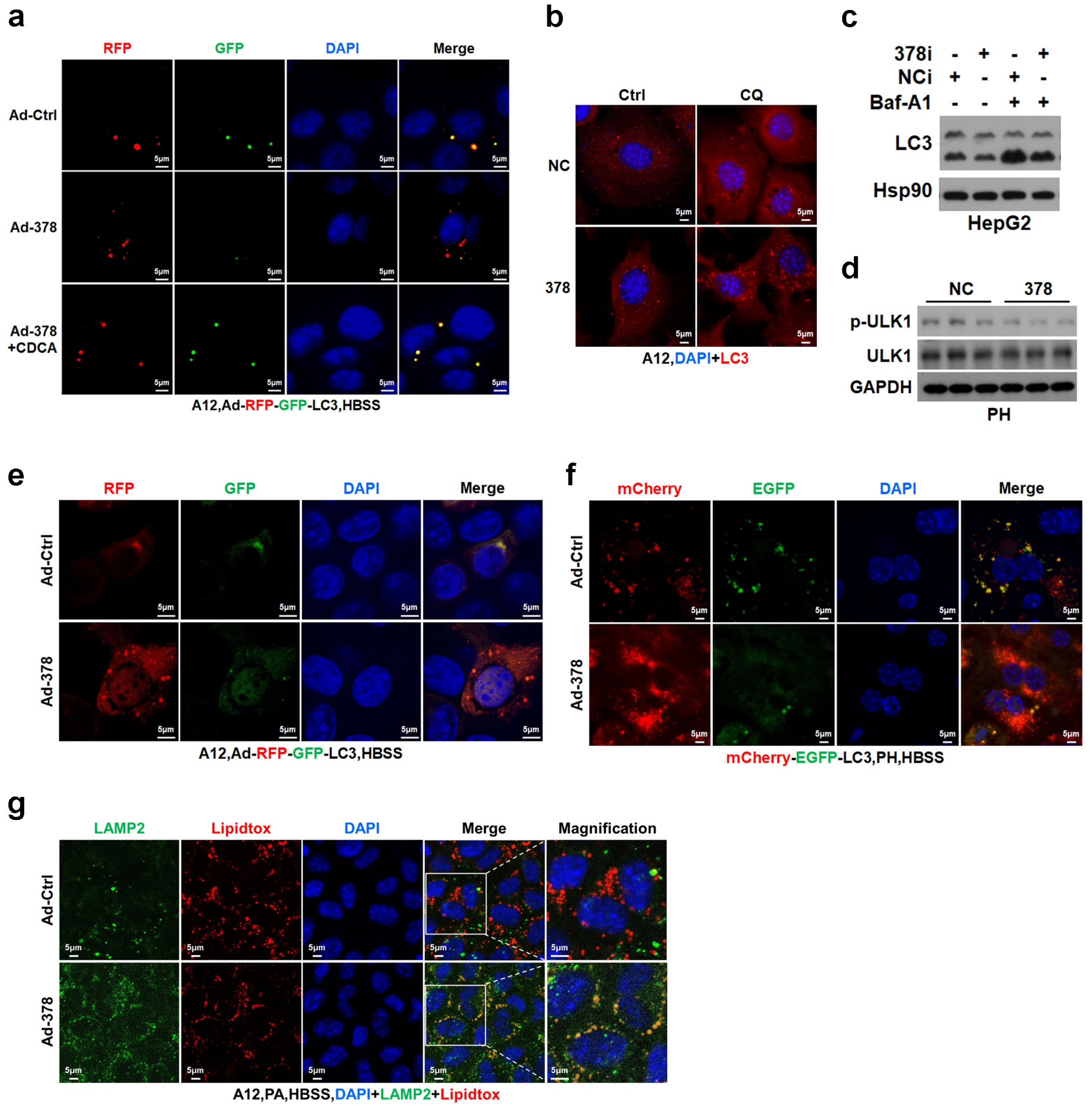

Supplementary Figure S4 related to Figure 4. (a) Ad-RFP-GFP-LC3-infected AML12 cells were treated with Ad-378 alone or Ad-378 together with CDCA, followed by 2 h of HBSS starvation. (b) Immunofluorescence staining of LC3 in AML12 cells transfected with miR-378 in the absence or presence of CQ. (c) Western blot analysis of LC3 in HepG2 cells transfected with miR-378 inhibitor in the absence or presence of Baf-A1. (d) Western blot analysis of p-ULK1 in PHs transfected with 378 mimics. (e) AML12 cells co-infected with Ad-378 and the LC3 autophagy reporter Ad-RFP-GFP-LC3, followed by HBSS treatment. (f) PHs from mCherry-EGFP-LC3 transgenic mice were infected with Ad- 378, followed by HBSS treatment for 2 h. (g) Immunofluorescence staining for Lipidtox and LAMP2 in PA-pretreated AML12 cells infected with Ad-378, followed by HBSS treatment for 2 h.

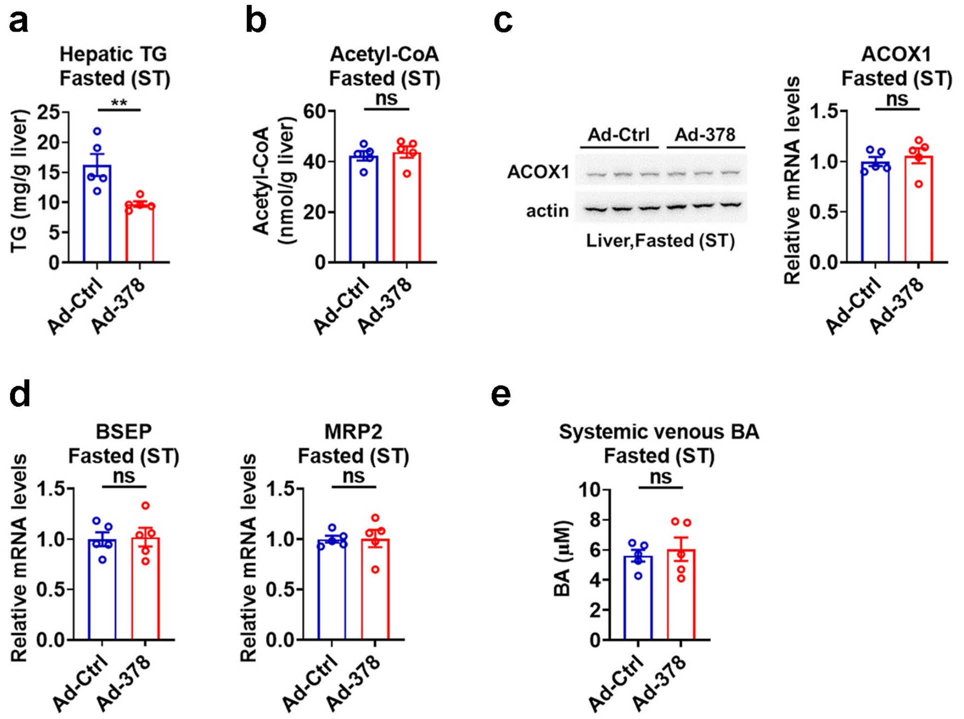

Supplementary Figure S5 related to Figure 5. (a) Liver TG levels in mice infected with Ad-378 after short-term fasting (n = 5). (b) Hepatic acetyl-CoA content in mice infected with Ad-378 after short-term fasting (n = 5). (c) Western blot analysis of ACOX1 protein and relative mRNA levels of ACOX1 in the livers of mice infected with Ad-378 after short-term fasting (n = 5). (d) Relative mRNA levels of FXR target genes (*BSEP* and *MRP2*) in the livers of mice infected with Ad-378 after short-term fasting (n = 5). (e) Systemic venous BAs in mice infected with Ad-378 after short-term fasting (n = 5). Means ± SEM are shown. P values were calculated by two-tailed unpaired Student's t-test. $^{*}P < 0.05$; $^{**}P < 0.01$; $^{***}P < 0.001$. ns, not significant.

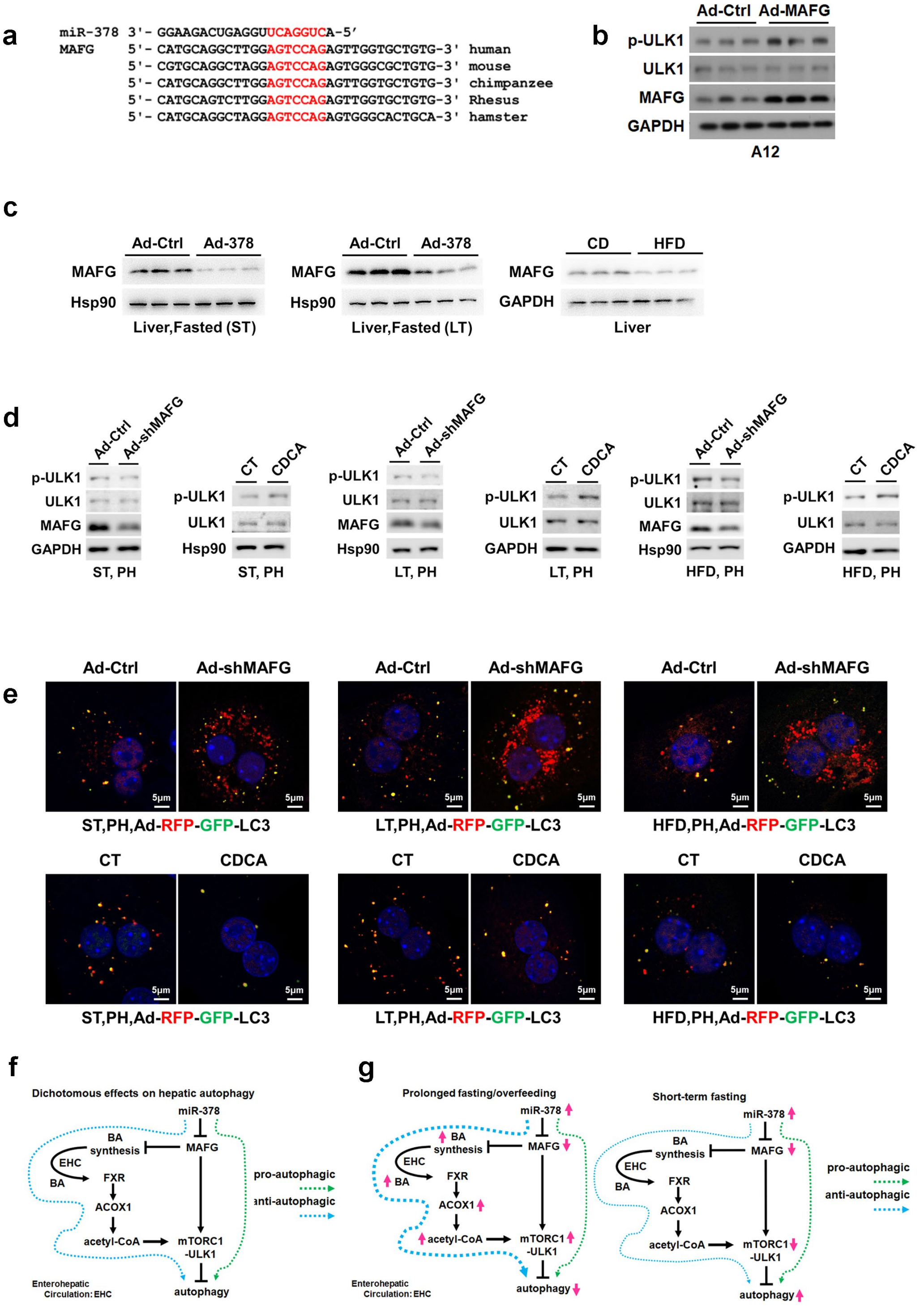

Supplementary Figure S6 related to Figure 6. (a) Sequence alignment of miR-378 and the 3'UTR of MAFG from various species. (b) Western blot analysis of p-ULK1 and MAFG in AML12 cells infected with Ad-MAFG. (c) Western blot analysis of MAFG in the livers of mice infected with Ad-378 after short-term fasting or long-term fasting and HFD-fed mice. (d) Western blot analysis of p-ULK1 in PHs infected with Ad-shMAFG or Ad-Ctrl, or treated with CDCA or vehicle control. PHs were

isolated from short-term fasted mice, long-term fasted mice, or HFD-fed mice. (e) Representative fluorescence images of PHs infected with the LC3 autophagy reporter Ad-RFP-GFP-LC3. PHs were infected with Ad-shMAFG or Ad-Ctrl, or treated with CDCA or vehicle control, and were isolated from short-term fasted mice, long-term fasted mice, or HFD-fed mice. (f and g) Schematic diagram of the working model of miR-378 and BAs in the regulation of hepatic autophagy.

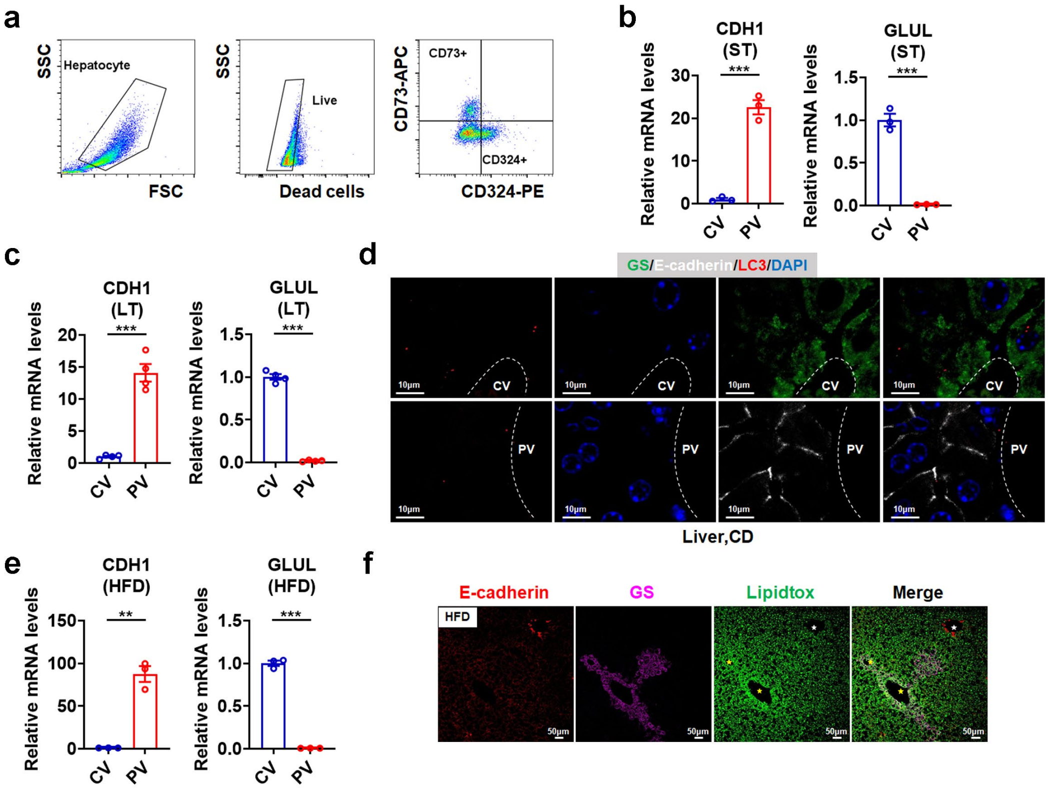

Supplementary Figure S7 related to Figure 7. (a) Gating strategy for isolating CD324^{+} (PV) and CD73^{+} (CV) hepatocytes. (b and c) Relative mRNA levels of CDH1 and GLUL in isolated CV and PV hepatocytes from fasted mice (*n* = 3, 4). (d) Immunofluorescent staining of LC3, E-cadherin, and GS in the livers of CD-fed mice. (e) Relative mRNA levels of CDH1 and GLUL in isolated CV and PV hepatocytes from HFD-fed mice (*n* = 3). (f) Immunofluorescent staining of LDs (Lipidtox), E-cadherin, and GS in the livers of HFD-fed mice. ST and LT stand for short-term and long-term, respectively. Means ± SEM are shown. *P* values were calculated by two-tailed unpaired Student's *t*-test. $^{**}P < 0.01$; $^{***}P < 0.001$.

Supplementary Table S1 Characteristics of the subjects with or without hepatic steatosis.

**Characteristic**	**Non-hepatic steatosis (*n* = 414)**	**Hepatic steatosis (*n* = 194)**	***P* value**
Age (years)	37 (30−44)	41 (34−47)	< 0.001
TG (mmol/L)	1.03 (0.79−1.30)	1.35 (1.04−1.70)	< 0.001
AST (U/L)	19 (16−21)	20 (18−23)	< 0.001
ALT (U/L)	17 (13−21)	23 (18−30.75)	< 0.001
Fasting blood glucose (mmol/L)	4.7 (4.5−5)	4.8 (4.6-5.1)	< 0.001
HDL-C (mmol/L)	1.36 (1.20−1.55)	1.21 (1.10−1.35)	< 0.001
LDL-C (mmol/L)	2.55 (2.16−2.95)	2.81 (2.41−3.09)	< 0.001
TC (mmol/L)	4.46 (3.98−4.87)	4.66 (4.22−4.94)	< 0.001
GGT (U/L)	18 (15−23.75)	26 (20−34) 5.2	< 0.001
HbA1c (%)	5.1 (4.9-5.28)	(5.1−5.4)	< 0.001

Data are presented as median (Q1–Q3).

Supplementary Table S2	Serum bile acid profiles of Ad-378 and Ad-Ctrl mice following long-term fasting									
Concentration (nmol/L)	Ad-Ctrl					Ad-378				
UDCA	134.222	182.898	175.081	155.577	139.381	122.037	108.728	119.380	128.967	135.179
HDCA	119.144	189.888	121.252	135.448	157.782	129.970	123.744	128.487	134.821	150.042
CDCA	113.102	165.342	142.414	126.788	122.941	141.146	117.201	131.990	139.934	167.107
DCA	783.208	930.576	751.102	785.350	839.126	1091.350	1049.800	1098.770	1096.890	1174.050
α-MCA	84.224	109.946	90.745	81.7914	87.013	140.907	126.580	137.538	141.197	176.726
β-MCA	1615.658	2482.080	1822.580	1599.614	1627.926	2183.760	2012.590	2153.370	2148.020	2387.130
CA	1156.090	1511.534	1434.758	1161.568	1094.694	5454.170	5164.310	5089.770	5543.820	6498.210
GCDCA	278.570	280.692	277.488	280.610	277.800	138.945	139.927	140.183	138.280	139.996
GCA	21.340	24.669	30.943	28.088	24.785	46.580	41.888	43.357	50.152	46.414
TUDCA	179.098	196.090	205.804	173.107	174.041	276.075	258.607	276.360	272.429	302.477
THDCA	83.174	86.719	92.355	83.358	85.832	128.609	118.757	125.768	135.247	153.496
TCDCA	197.876	228.028	200.092	190.074	200.988	130.141	128.951	138.194	132.679	155.812
TDCA	458.714	496.188	413.382	460.682	461.124	1028.310	1033.720	995.487	1047.090	1011.830
T-α-MCA	979.110	1145.994	1114.204	961.204	991.138	1216.960	1138.820	1265.690	1241.230	1443.380
T-β-MCA	2266.880	2723.680	2675.280	2357.240	2369.920	6231.500	5869.980	6030.740	6201.930	6541.970
TCA	5001.220	5936.180	5945.020	5491.320	5030.020	19256.800	18449.100	18925.900	18680.100	21109.100

Supplementary Table S3 Sequence information for primers (5'−3').

Name	Sequence	Note
mACOX1-F	CATATGACCCCAAGACCCAAG	RT-PCR
mACOX1-R	CATGTAACCCGTAGCACTCC	RT-PCR
mSHP-F	CGATCCTCTTCAACCCAGATG	RT-PCR
mSHP-R	AGGGCTCCAAGACTTCACACA	RT-PCR
mBSEP-F	CCAGGAAAAGCATGTGTGAA	RT-PCR
mBSEP-R	CGACAGCAATTCCAGCATAG	RT-PCR
mMRP2-F	GTGTGGATTCCCTTGGGCTTT	RT-PCR
mMRP2-R	CACAACGAACACCTGCTTGG	RT-PCR
mACLY-F	TTCGTCAAACAGCACTTCC	RT-PCR
mACLY-R	ATTTGGCTTCTTGGAGGTG	RT-PCR
mACSS2-F	GCTTCTTTCCCATTCTTCGGT	RT-PCR
mACSS2-R	CCCGGACTCATTCAGGATTG	RT-PCR
mPDK4-F	AGGGAGGTCGAGCTGTTCTC	RT-PCR
mPDK4-R	GGAGTGTTCACTAAGCGGTCA	RT-PCR
mCDH1-F	TGGGCAGAGTGAGATTTGA	RT-PCR
mCDH1-R	CCACTTTGAATCGGGAGTCT	RT-PCR
mGLUL-F	CCACCGCTCTGAACACCTT	RT-PCR
mGLUL-R	TGGCTTGGACTTTCTCACCC	RT-PCR
18s-F	ACCGCAGCTAGGAATAATGGA	RT-PCR
18s-R	CAAATGCTTTCGCTCTGGTC	RT-PCR
miR-378-RT	CTCAACTGGTGTCGTGGAGTCGGCAA TTCAGTTGAGCTTCTGAC	stem-loop RT
U6-RT	CTCAACTGGTGTCGTGGAGTCGGCAA TTCAGTTGAGATATGGAA	stem-loop RT
miR-378-F	ACACTCCAGCTGGGACTGGACTTGGA	stem-loop PCR
U6-F	ACACTCCAGCTGGGATTCGTGAAGCG	stem-loop PCR
Universal-R	CTCAACTGGTGTCGTGGAGTCGG	stem-loop PCR
ACOX1-F	GACGGACGAATCCCCTGC	ChIP-PCR
ACOX1-R	GTGAACCTGGAGACGTGAGG	ChIP-PCR
